# Impact of *Fusarium* Infection on Potato Quality, Starch Digestibility, In Vitro Glycemic Response, and Resistant Starch Content

**DOI:** 10.3390/jof9040466

**Published:** 2023-04-12

**Authors:** Rahul Kumar Tiwari, Milan Kumar Lal, Ravinder Kumar, Sanjeev Sharma, Vinay Sagar, Awadhesh Kumar, Brajesh Singh, Rashmi Aggarwal

**Affiliations:** 1 Division of Plant Protection, ICAR—Central Potato Research Institute, Shimla 171001, India; 2 Division of Plant Pathology, ICAR—Indian Agriculture Research Institute, New Delhi 110012, India; 3 Division of Crop Physiology, Biochemistry and Postharvest Technology, ICAR—Central Potato Research Institute, Shimla 171001, India; 4 Division of Crop Physiology and Biochemistry, ICAR—National Rice Research Institute, Cuttack 753006, India

**Keywords:** Lesion index, glycemic load, starch, dry rot, *Fusarium sambucinum*, tuber

## Abstract

Potato dry rot disease caused by multiple *Fusarium* species is a major global concern in potato production. In this investigation, the tubers of cultivars Kufri Jyoti and Kufri Frysona were artificially inoculated with an individual or combined inoculum of *Fusarium sambucinum* and *Fusarium solani*. *Fusarium sambucinum* caused a significantly higher lesion development (*p* < 0.01) than *Fusarium solani,* irrespective of cultivars. The combined inoculum of both the *Fusarium* species caused significantly higher rot development (*p* < 0.005) in inoculated tubers. Analyses of starch and amylose content revealed that individual or mixed infection of fungi caused a significant reduction (*p* < 0.005) in these parameters compared to healthy tubers. The increased starch digestibility due to fungal infection caused a higher glycemic index and glycemic load. The resistant starch also deteriorated in the infected potato tubers as compared to the control. Kufri Jyoti showed a higher starch and amylose content reduction in response to the treatments compared to Kufri Frysona. The correlation analysis demonstrated a negative correlation in lesion diameter and rot volume with starch and amylose content (*p* < −0.80). However, the glycemic index and resistant starch were positively correlated with lesion development. Altogether, these findings highlight the progressive deterioration of quality parameters, which will be a critical concern for processing industry stakeholders and consumers.

## 1. Introduction

Potato (*Solanum tuberosum* L.) is the foremost cash crop globally consumed by more than one billion people in 150 countries [1]. In developing nations, the crop has been designated as an integral source of food, employment, and occupation. Three leading countries in global potato production include China, India, and Russia, which produced 99, 43.8, and 31.1 million tons of potato, respectively, in the year 2020 [2]. With the huge demand from the growing population and processing industries, there is enormous pressure for higher production. At the same time, minimization of losses due to biotic and abiotic stresses is crucial in potato cultivation. Potato is a vegetatively propagated underground vegetable crop; therefore, the chances of soil and tuber-borne diseases are always high. Globally, more than 40 soil-borne diseases were reported to hamper potato production by damaging the tuber, which is the most economically important part of the plant [3,4]. 

During the past decade, potato dry rot disease, which is reported to be caused by several *Fusarium* species, has emerged as a critical soil- and tuber-borne problem in potato cultivation [5]. The disease affects the standing crop by causing *Fusarium* wilt, but severe problems arise in cold storage, where the tuber rot conditions appear due to *Fusarium* infection in healthy tubers. Worldwide, more than 13 pathogenic species of *Fusarium* are reported to be associated with this disease [6]. *Fusarium sambucinum* is the most aggressive species responsible for dry rot disease, as reported by China, India, Algeria, and several other potato growing regions [7]. Numerous *Fusarium* species have been documented from Iran as causal agent of potato dry rot, highlighting *Fusarium sambucinum* and *Fusarium solani* as the predominant species. *Fusarium solani*, *Fusarium oxysporum* f. sp. *tuberosi*, *Fusarium sambucinum*, and *Fusarium graminearum* were observed to be more frequent in Tunisian potato cultivars [8]. In Great Britain, the major species responsible for dry rot was *Fusarium coeruleum* (Libert) [9]. The common cereal fungus *Fusarium graminearum* is also frequently reported to cause dry rot of potato in the states of North Dakota and Michigan in the United States. Recently *Fusarium proliferatum* has been reported for the first time as the causal agent of dry rot disease in Indian potato cultivars. *Fusarium sambucinum*, *Fusarium oxysporum*, and *Fusarium solani* are intercepted most frequently in potato cold storage in rot-affected tubers [10].

The losses to the dry rot-affected tubers have been reported as both quantitative and qualitative [11,12]. The quantitative loss in terms of yield has been previously reported to be as high as 25–60% in cold storage of potato [12,13,14]. The average economic loss of USD 100–250 million was reported due to potato dry rot disease in the United States [5]. Likewise, in a specific province in China, 88% of total post-harvest loss in potato was due to infection of *Fusarium sambucinum* and *Fusarium solani* [13]. A previous record revealed that 50% of tuber lots in storage units in Michigan were infected with *Fusarium* species [14]. In India, 15–22% of potato dry rot disease incidences have been documented in various potato-growing regions [15]. Reports of qualitative loss to the tubers due to dry rot disease are limited. Recently, it was reported that *Fusarium sambucinum* and *Fusarium oxysporum* severely affect nutritional quality parameters such as sucrose, soluble sugars, amylose, and amylopectin [16]. Additionally, the total carbohydrates and reducing sugars were also hampered under dry rot infection. Some recent reports highlighted that *Fusarium oxysporum* infection in potato leads to an upsurge in reducing sugar and a reduction in starch content in tubers [5]. Some microbes, such as *Microbacterium aurum* strain B8, have been suggested to synthesize extracellular starch hydrolysing enzyme, which is responsible for the deterioration of potato wheat and tapioca starch [17]. There are reports of other biotic stresses that can affect potatoes, such as viral infections, which have been documented along with their impact on the nutrient levels in the tubers. The potato apical leaf curl infection in tubers of cultivars Kufri Pukhraj showed a significant reduction in starch, amylose, soluble sugars, and total carbohydrates [13,17,18]. 

The assessment of starch quality-related parameters and other nutritional components of the tuber is crucial from the viewpoint of processing quality attributes of tubers [19,20,21]. The upsurge in reducing sugar content and dry matter declination negatively affects the processing quality attributes of the tubers that hamper the formation of chips, French fries, and post-harvest traits [22,23]. The increased sucrose, reducing sugars under biotic and abiotic stresses, not only affects the cooking quality but also leads to the formation of acrylamide, which is non-desirable for consumption [24,25,26].

Biotic infections, as well as abiotic stress, can affect the starch component of potatoes, leading to changes in the glycemic index (GI) and glycemic load (GL) of starchy food products [27]. Potato comes under the category of high GI foods, and it is usually recommended to eat in food combinations with low GI foods [22,27]. Resistant starch (RS) is considered a good dietary fiber source in potato for improved gut microbiota, a balanced GI, and a strengthened digestive tract [26,28]. The RS is negatively correlated with GI since RS slows down digestion, resulting in low glucose absorption in the bloodstream. A previous report suggests that starchy food crops under the influence of biotic stress such as drought have high GL and low RS [29]. Reports of fungal pathogen infection-mediated alterations in GI and RS of potato tubers are not available to the best of our knowledge. Here we have highlighted the impact of *Fusarium* infection on disease progression along with changes in starch, amylose, GI, and RS in two cultivars (Kufri Jyoti and Kufri Frysona). The outcome will assist future studies with mechanistic insights of alterations in nutritional components of potato tubers and the resultant effect on consumer health.

## 2. Materials and Methods

### 2.1. Maintenance of Fungal Pure Culture and Potato Cultivars

The potato tubers of cultivars Kufri Jyoti and Kufri Frysona were obtained from ICAR–Central Potato Research Institute, Regional Station, Modipuram, India. For all these experiments, it was ensured that the tubers were freshly harvested, uniform in size, and free from adhering fungal spores or dust particles. The tubers were washed with distilled water and dried in the shade followed by surface sterilization with 1% sodium hypochlorite solution and drying. The fungal pathogen in the current experiment was isolated from the dry rot-infected tuber samples collected from the Himachal Pradesh and Madhya Pradesh regions. Pathogen isolation was carried out on the tubers exhibiting dry rot symptoms. In a laminar cabinet, the diseased and healthy (1 × 1 mm) parts were excised from the infected tubers and surface sterilized with 1% sodium hypochlorite for 1 min, 70% ethanol for 30 s, and washed with distilled water. The sterilized part was kept in potato dextrose agar media (HiMedia Biosciences, Mumbai, India) and incubated at 18 to 24 °C. After two days, the initiating fungal growth from the diseased part was carefully transferred to fresh PDA plates to establish the pure culture of the fungi. 

### 2.2. Morphological and Molecular Identification of the Fungus

The completely grown pure cultures of fungi were observed under a compound microscope, and parameters such as colony morphology, presence of aerial mycelium, macroconidia and microconidia characteristics, length and breadth index of spores, and chlamydospore morphology were examined [30]. Additionally, Koch’s postulates were also confirmed by the pathogenicity test of both the isolates on cultivars Kufri Jyoti and Kufri Frysona. Once the morphology and pathogenicity were established, the pure culture was mass multiplied in potato dextrose broth for 5 days (HiMedia Biosciences, Mumbai, India). The mycelial balls were filtered and dried, and genomic DNA was extracted using the CTAB method. The quality of the DNA was assessed using both NanoDrop and agarose gel techniques. Following this, a PCR was carried out using 50 ng/μL DNA. Each 20 μL reaction included 10 μL of Taq buffer, 1 μL of both forward and reverse primers, 2 μL of template DNA, and 6 μL of nuclease-free water to adjust the final volume. The fungus-specific primer translation elongation factors 1 alpha EF1Fα ATGGGTAAGGAAGACAAGAC and EF1Rα GGAAGTACCAGTGATCATGTT were used for the amplification of purified DNA. The reaction set-up had an initial denaturation of 4 min at 95 °C, 33 cycles at 95 °C for 30 s, 55 °C for 1 min, 72 °C for 1 min, and a final extension at 72 °C for 10 min. The observation of the amplicon was performed with gel electrophoresis on 1.0% agarose gel with a TBE running buffer stained with ethidium bromide. The gel documentation (INTAS, Homburg, Germany) captured gel images. The amplicons visible under UV light were carefully excised from the gel. The gel extraction was executed using a Qiagen kit as per the manufacturer’s protocol. The DNA quality was further checked with NanoDrop and a gel run on agarose. Two isolates were sent for direct sequencing (Eurofins Genomics India Pvt. Ltd., Bengaluru, India). A BLAST search was carried out for sequenced data using the NCBI database. The sequences were submitted to the NCBI database, and accession numbers were obtained for both isolates. 

### 2.3. Tuber Inoculation and Estimation of Lesion Diameter, Lesion Depth, and Rot Volume

#### 2.3.1. Inoculum Preparation

Two pathogenic isolates, namely *Fusarium sambucinum* HF9 and *Fusarium solani* MP5, which were identified through morpho-molecular methods, were used in this disease evaluation experiment. On PDA culture plates, the pure culture of these two isolates was already established. By taking a mycelial bit, it was subsequently cultured in potato dextrose broth (PDB) to make spore suspensions. The cultures were filtered through a layer of cheesecloth to remove bulky mycelium and media particles and then passed through Whatman No. 1 filter paper after incubation for 7 days at 18 °C. Using a hemacytometer, the final spore concentration in the suspension was adjusted to 2.5 × 10^6^ conidia/ml. Tuber inoculation was performed with an equal volume of inoculum (50 μL). The three treatments comprised *Fusarium sambucinum* (FSam), *Fusarium solani* (FSol), and mixed inoculum (FSam + FSol) along with water agar inoculated control tubers. 

#### 2.3.2. Tuber Inoculation

These freshly harvested Kufri Jyoti and Kufri Frysona tubers were surface sterilized for 1 minute with 70% ethanol and further rinsed with 1% sodium hypochlorite (NaOCl) for 1 minute before being washed again with sterile distilled water. Using a sterile cork borer in a laminar cabinet, a uniform-size wound that measured 6 mm in diameter and 6 mm in depth was created on the tuber surface. The inoculum (FSam, FSol, FSam + FSol) was injected into each uniformly sized wound (50 μL each). Tubers were simultaneously wounded in the control group, but water agar (WA) was applied to the wounds rather than the *Fusarium* spore suspension. A sterile paraffin wax strip was applied to each treated wound. The estimation of disease-related parameters in this experiment employed twenty tubers per isolate, and each experiment was repeated three times. The growth chamber facility at the ICAR–Central Potato Research Institute, Shimla, was utilized to store the tubers, which were kept in a plastic cabinet. The temperature and relative humidity of the storage unit were maintained at 18 °C and 90%, respectively.

#### 2.3.3. Periodic Disease Assessment

After successful inoculation and incubation in the growth chambers, the tubers of each cultivar were periodically evaluated for the development of lesion diameter, lesion depth, and rot volume over the duration of two months (0 days, 20 days, 40 days, 60 days). The previously used methodology was referred to in this experiment [31]. Four perpendicular diameters of the lesion were recorded to determine the mean diameter on each site of inoculation. Tubers were cut into two halves of the inoculation sites along the longitudinal axis. The inward progression of the lesion was also recorded with three perpendicular readings of lesions to calculate the mean lesion depth. The rot volume was calculated using the formula:

V = πr^2^ h/3, where V is volume, r is half the lesion diameter, and h is the depth of the lesion.

### 2.4. Tuber Processing for Biochemical Analysis

The potato tubers cultivars (Kufri Jyoti and Kufri Frysona) were processed for biochemical analysis. Again, the fungus inoculated tubers and control (water agar inoculated) tubers were used for biochemical analysis upon initial, 20 days, 40 days, and 60 days post-inoculation. The potato tubers were ground to make a paste and subsequently lyophilized in a freeze dryer (Scanvac Cool Safe, Labogene, Lynge, Denmark). Moreover, the freeze-dried samples were pulverized into powdered form and passed through a 100-mesh size sieve. All the analyses were executed in triplicate. 

#### 2.4.1. Determination of Total Starch Content

The total starch content in Kufri Jyoti and Kufri Frysona tubers was determined according to a previously standardized method [32]. The analysis was performed in dried potato powder of control (water agar inoculated, non-infected), FSam, Fsol, and FSam + FSol infected potato tubers of Kufri Jyoti and Kufri Frysona. We suspended 100 mg of the sample in 6.5 mL of 52% perchloric acid and 5 mL of distilled water. The suspension was then incubated at room temperature (25 °C) for 24 h. After incubation, we centrifuged the samples, and the residue was extracted with an additional 6.5 mL of 52% perchloric acid. We then centrifuged the mixture again and combined both supernatants, adjusting the final volume to 50 mL with distilled water. Next, we prepared a mixture of 50 µL of the sample and 950 µL of distilled water and added 2 mL of anthrone-sulfuric acid reagent to it. The reagent was prepared by dissolving 200 mg of anthrone in 100 mL of chilled, concentrated sulfuric acid. We boiled the samples for 8 min and then allowed them to cool to room temperature. Finally, we measured the optical density of the samples at 620 nm using a T60 Visible Spectrophotometer (PG Instruments, New Delhi, India). The units were expressed in g kg^−1^ on a dry weight basis.

#### 2.4.2. Determination of Amylose Content

The amylose content in both cultivars (Kufri Jyoti and Kufri Frysona) was measured by colorimetric estimation according to the method described by Juliano et al. using the amylose–iodine complex [33]. Once the color was developed, the absorbance was determined at 620 nm using a UV/vis spectrophotometer (Model-4001/A, Thermo Spectronic, Waltham, MA, USA). The units of amylose were expressed as a percentage. 

### 2.5. Determination of Glycemic Index (GI), Glycemic Load (GL), and Resistant Starch (RS)

#### 2.5.1. Estimation of In Vitro GI

The GI of inoculated (FSam, FSol, FSam + FSol) and control (water agar inoculated) tubers was estimated according to the in vitro method [34]. For the starch digestibility experiment, Pepsin (3000 U/mL, from porcine gastric mucosa, Sigma–Aldrich, St. Louis, MO, USA), α-amylase (10 U/mg solid, porcine pancreatic, Sigma–Aldrich, St. Louis, MO, USA), and amyloglucosidase (3300 U/mL, HiMedia Biosciences, Mumbai, India) were used as digestive enzymes. About 0.2 g of potato powder was boiled with 2 mL of distilled water for 2 minutes in a boiling tube. Subsequently, 5 mL of 0.1 M phosphate buffer (pH 6.9) (MP Biomedicals, Santa Ana, CA, USA) was added along with vigorous shaking. Prior to the addition of 0.2 mL of pepsin (250 mg/mL), the pH of the solution was brought down to 2.5 with o-phosphoric acid (Merck, Darmstadt, Germany) and placed in an incubator shaker at 37 °C and 120 rpm for 60 min. The pH was finally adjusted to 6.9 with potassium hydroxide (20%), and 0.3 mL of α-amylase (125 mg/mL) was added. Next, the solution was transferred to a dialysis membrane (50 width: 24.26 mm, diameter: 14.3 mm, HiMedia Biosciences, Mumbai, India, Catalog no. LA387-5MT) 10 cm in length, which was placed in a 50 mL centrifuge tube containing 40 mL of 0.1 M phosphate buffer (pH 6.9). The setup was incubated at 37 °C and 120 rpm for 180 min.

At every 30 min interval, 0.5 mL of aliquots were drawn from the centrifuge tube, up to 3 h, and mixed with 1.5 mL of 0.4 M sodium acetate buffer (pH 4.75), followed by the addition of 40 µL of amyloglucosidase and incubation at 50 °C for 30 min. The final volume was 10 mL with distilled water. An aliquot of 0.3 mL (in triplicate) was incubated with GOPOD (glucose oxidase–peroxidase) at 50 °C for 30 min. D-glucose (0.2 g) (MP Biomedicals, USA) was used as a standard carbohydrate for this experiment. The average value was calculated and used to plot curves followed by calculating the area under the curve (AUC) of starch digestibility. The starch hydrolysis index (SHI) was calculated by dividing the AUC of the potato sample by the AUC of glucose and expressed as a percentage. 

The starch hydrolysis index was calculated by dividing the area under the curve (AUC) of the treated samples by the AUC of glucose (standard). The main formula used for in vitro estimation of GI was:

GI = 39.21 + (0.803 × Starch Hydrolysis Index).

#### 2.5.2. Estimation of In Vitro GL

A 100 g of potato sample from respective treatments was used to estimate the GL using the formula:

GL = (GI × Available carbohydrate)/100.

#### 2.5.3. Determination of RS

The RS for inoculated (FSam, FSol, FSam + FSol) and control (water agar inoculated) tubers was recorded according to the previously established protocol [35,36]. To estimate the resistant starch (RS) content in the sample, the lyophilized sample was first digested with 5 mL of pancreatic 𝛼-amylase (10 mg mL^−1^) that contained amyloglucosidase (300 U mL^−1^). The mixture was incubated in a 25 mL conical flask at 37 °C with continuous shaking at 200 shakes per minute for 16 hours. The enzymatic reaction was then terminated using ethanol, and centrifugation was performed to separate non-RS components. The supernatant was discarded, and the remaining pellet was air-dried before being resuspended in 2 M potassium hydroxide (KOH). The mixture was then stirred in an ice bath for 25 min. Next, 8 mL of 1.2 M sodium acetate at pH 4.3 was added, followed by 0.1 mL of amyloglucosidase (3300 U mL^−1^). The mixture was then incubated at 50 °C for 30 min. The final color was developed by mixing a 0.1 mL aliquot with 3 mL of the GOPOD reagent and incubating at 50 °C for 20 min. The final absorbance was measured at 510 nm using a UV/vis spectrophotometer and expressed as a percentage (%) of dry matter. 

### 2.6. Statistical Analysis

All the parameters evaluated in this study were done in triplicate (n = 3). The data analysis was performed using SAS (version 9.0, SAS Institute Inc., Cary, NC, USA) and Prism 6.01 (GraphPad Software Inc., San Diego, CA, USA). The significant differences in mean lesion diameter, depth, and rot volume were estimated with two-way ANOVA and Tukey multiple comparisons tests (*p* < 0.05). 

## 3. Results 

### 3.1. Morphological Identification of Fungal Isolates

**Figure 1:**
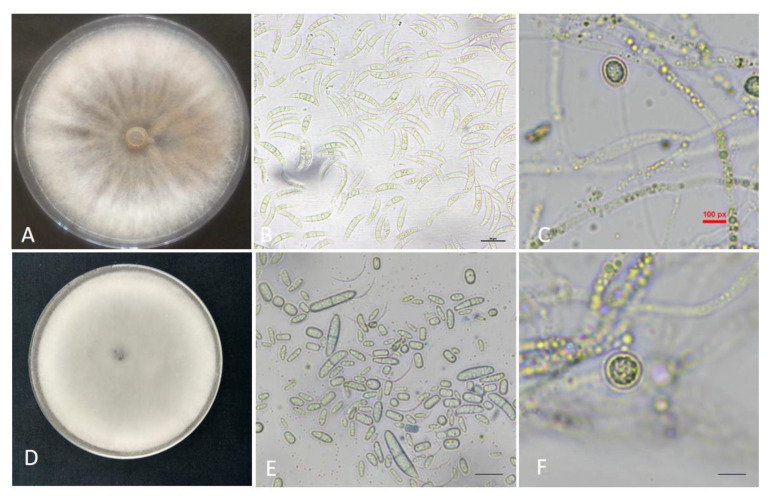
The morphological characteristics of *Fusarium sambucinum* and *Fusarium solani* (**A**) Pure culture *Fusarium sambucinum* on petri plate; (**B**) Macroconidia of *Fusarium sambucinum*; (**C**) Chlamydospores (**D**) Pure culture *Fusarium solani* (**E**) Microconidia and macroconidia (**F**) Chlamydospores. The fungal spores (**B**,**C**,**E**,**F**) were observed under a compound microscope at 40 X magnification (scale bar: 100 µm).

The etiology of potato dry rot in the samples collected from Himachal Pradesh and Madhya Pradesh, India, was established through morpho-molecular identification with a clear aim of species-level identification of the fungus involved in the disease development. The Madya isolate MP5 was observed to have swiftly grown rusty orange fungal colonies, apparent concentric rings, and an enormous mass of macroconidia (Figure 1A). The occurrence of aerial mycelium either singly or in small false heads and monophialides were easily identifiable features. The abundant macroconidia were short, 3–5 septate, relatively slim, apically pointed, and foot-shaped basal cells (Figure 1B). The microconidia were not found in cultures, but chlamydospores were observed in the solitary form (Figure 1C). The fungal infection on dry rot-affected tubers with this isolate showed a slight tan-brown appearance. The morphological characteristics were similar to *Fusarium sambucinum* as per the fusarium laboratory reference manual [37]. 

Pure cultures of fungal isolate (HF9) identified in the samples collected from Himachal Pradesh often had white to cream sparse mycelium. Creamish white sporodochia were produced in abundance (Figure 1D). Conidiophores were shown to have either singly aerial mycelium or less frequent false heads. Macroconidia were equitably wide, straight, stout, and thin at the apical cell. Frequently oval (0–1 septate) microconidia were also observed in these cultures (Figure 1E). Additionally, the solitary chlamydospores were evident in thirty-day-old cultures (Figure 1F). The morphological features resembled the characteristics of *Fusarium solani* [37].

### 3.2. Molecular Identification and Phylogenetic Analysis

The morphological characterization of *Fusarium* isolates was further supplemented with molecular studies. The sequence of isolate HF9 matched 99–100% with *Fusarium solani* strain A8r4 (MK560287.1) and *Fusarium solani* strain Zb-RR (MZ419443.1). Likewise, the sequence of isolate MP5 showed 99–100% similarity with *Fusarium sambucinum* isolate UN1MNE (MK358119) and *Fusarium sambucinum* isolate T1Tiar7 (MK752459). The two isolates in this study were identified as *Fusarium sambucinum* MP5 and *Fusarium solani* HF9, and the respective sequences were submitted to GenBank with accession numbers OM190496.1 and OM190518.1.

### 3.3. Effect of Dry Rot Infection on Lesion Diameter 

**Figure 2:**
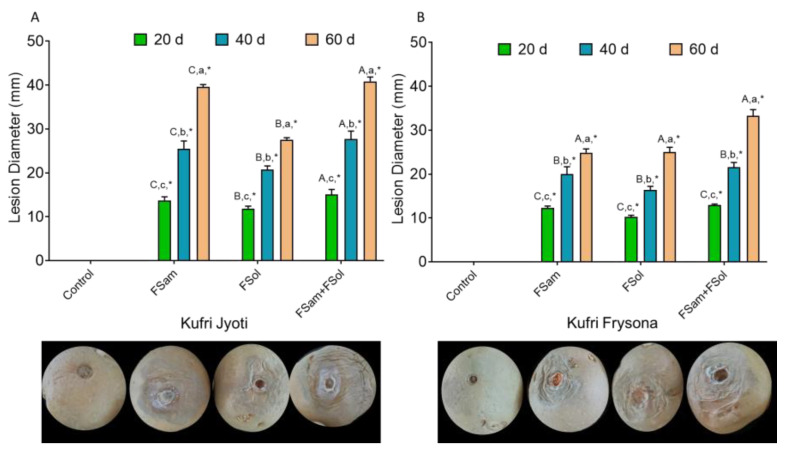
The effect of dry rot infection on lesion diameter on (**A**) Kufri Jyoti (**B**) Kufri Frysona; control, no pathogen infection; FSam, *Fusarium sambucinum*; FSol, *Fusarium solani*; FSam + FSol, *Fusarium sambucinum* + *Fusarium solani*. Capital letter = significance of inoculum (within one date/cultivar combo); Lower case letter = significance of date (within one inoculum/cultivar combo); * = significance between cultivars (within one inoculum/date combo).

The temporal evaluation of dry rot lesions on tubers inoculated with FSam, FSol, and FSam + Fsol over the period of two months revealed the typical symptoms of dry rot in Kufri Jyoti and Kufri Frysona. The clearly observable symptoms in terms of lesion development were evident at the inoculation site. The necrotic tan brown to black lesions with clear margins were observed after 20 days. The skins appeared wrinkled and shriveled. After 40 and 60 days, the lesions had covered all the surfaces of the inoculated tubers with the mummification of inoculated tubers. Symptom severity was higher in Kufri Jyoti as compared to Kufri Frysona in all three treatment combinations of FSam, FSol, and FSam + FSol. The control tubers were symptomless, and the wound was dry at its location without showing any necrotic lesions. The observation of mean lesion diameter (MLD) on FSam, FSol, and FSam + FSol inoculated tubers showed significant differences (*p* < 0.001) based on cultivar type, treatment combination, and duration of storage after inoculation. A consistent increase in lesion diameter was recorded in both cultivars after 20, 40, 60 days; however, the average lesion diameter was greater in Kufri Jyoti as compared to Kufri Frysona in all the challenge inoculations of the individual (FSam, FSol) and mixed infections (FSam + FSol) (Figure 2A,B and Tables S1 and S2). In Kufri Jyoti, the MLD was 13.7 mm, 25.5 mm, and 39.6 mm after 20, 40, and 60 days of inoculation of FSam, while it was 11.8 mm, 20.8 mm, and 27.5 mm in FSol inoculated tubers. Interestingly, the mixed infection (FSam + FSol) caused significantly higher lesion development with MLD 15.06 mm, 27.7 mm, and 40.8 mm. 

The results depict a higher lesion diameter from *Fusarium sambucinum* as compared to *Fusarium solani*; at the same time, the combined infection caused the higher loss in terms of lesion development. The pattern of lesion diameter progression was similar in the mean lesion diameter (MLD) values of FSam, FSol, and FSam + FSol inoculated Kufri Frysona tubers. Here too *Fusarium sambucinum* caused more extensive lesions followed by FSam + FSol.

### 3.4. Estimation of Lesion Depth in Inoculated Tubers

**Figure 3:**
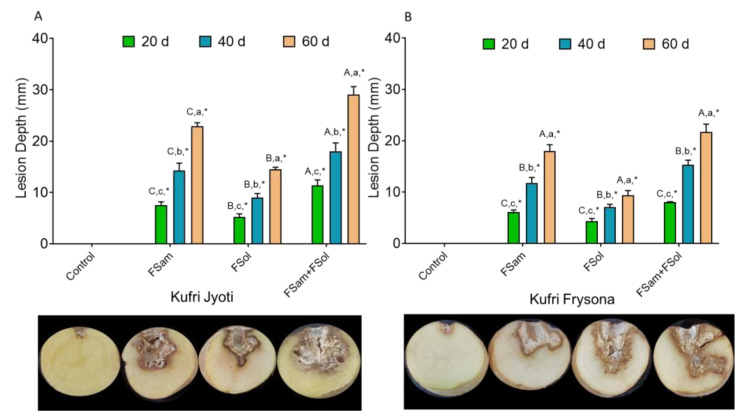
The figure represents the effect of dry rot infection on lesion depth on (**A**) Kufri Jyoti (**B**) Kufri Frysona; control, no pathogen infection; FSam, *Fusarium sambucinum*; FSol, *Fusarium solani*; FSam + FSol, *Fusarium sambucinum* + *Fusarium solani*. Capital letter = significance of inoculum (within one date/cultivar combo); Lower case letter = significance of date (within one inoculum/cultivar combo); * = significance between cultivars (within one inoculum/date combo).

In all tubers inoculated with individual (FSam or FSol) or combined (FSam + FSol) spore suspensions, the lesion progressed internally, and significant differences were observed based on cultivar type, duration of storage, and species responsible for the rot development. In cultivar Kufri Jyoti, the combined infection (FSam + FSol) caused a mean lesion depth ranging from 11.4 mm after 20 days to 29.1 mm after 60 days, while the FSam infection caused a lesion depth of 7.5 mm after 20 days to 22.9 mm after 60 days. A shallower lesion depth was evident in FSol-inoculated tubers, showing 5.2 mm and 14.5 mm, respectively, at 20 and 40 days. Tukey’s multiple comparison analysis depicted significant differences (*p* < 0.05) among this observation of the depth of fungal penetration both in the cultivars and among the fungal treatments (Figure 3A,B, Tables S3 and S4). The mean depth of lesions in Kufri Frysona was 18.03, 9.4, and 21.7 in respective FSam, FSol, and Fsam + Fsol treated tubers after 60 days of inoculation. The pattern of lesion depth has shown a significant correlation with lesion diameter, and it was clearly observed that *Fusarium sambucinum* was more penetrative than *Fusarium solani*. However, when combined inoculation was given to the tubers, the fungi showed increased interaction and caused greater loss to the tuber as compared to the control.

### 3.5. Association of Fusarium Infection and Rot Volume 

**Figure 4:**
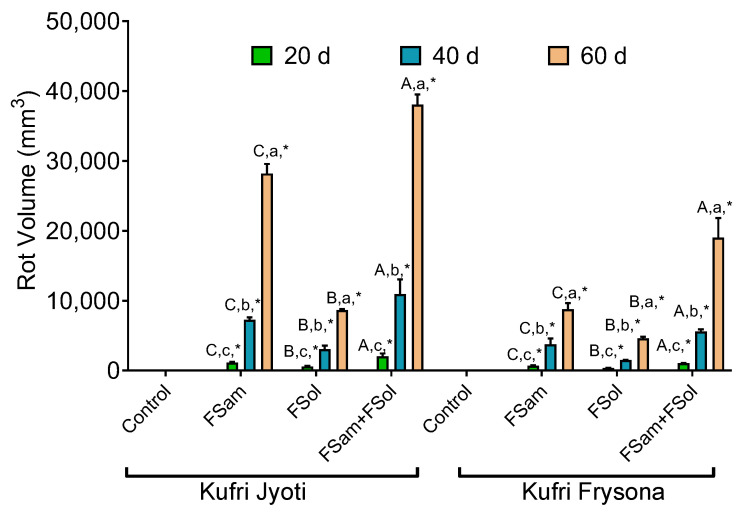
Effect of dry rot infection on rot volume after 0, 20, 40, and 60 d of infection. Scale in the figure denotes rot volume expressed in mm^3^. Control, no pathogen infection; FSam, *Fusarium sambucinum*; FSol, *Fusarium solani*; FSam + FSol, *Fusarium sambucinum* + *Fusarium solani*. Capital letter = significance of inoculum (within one date/cultivar combo); Lower case letter = significance of date (within one inoculum/cultivar combo); * = significance between cultivars (within one inoculum/date combo).

The volume of rot varied significantly (*p* < 0.05; Table S5) among cultivars Kufri Jyoti and Kufri Frysona. The highest rot volume was recorded in FSam + FSol inoculated potatoes compared to inoculation of individual fungus (either FSam or FSol) (Figure 4). A temporal rise in tuber rot volume was also recorded in individual (either FSam or FSol) and combined (FSam + FSol) inoculation in both Kufri Jyoti and Kufri Frysona. In FSam inoculated tubers of cultivar Kufri Jyoti, the volume of rot increased in significantly higher proportion than FSam inoculated tubers of Kufri Frysona. Likewise, the rot volume was proportionately higher in FSam + FSol inoculated Kufri Jyoti cultivars compared to Kufri Frysona. Altogether, keeping in mind the observations of lesion diameter and lesion depth, and correlating those with rot volume, the higher susceptibility of Kufri Jyoti was evident as compared to Kufri Frysona. Higher aggressiveness and disease index through combined infections was also apparent as compared to individual species infections, followed by greater pathogenicity of FSam. 

### 3.6. Starch and Amylose Content in Dry Rot Infected Tubers 

**Figure 5:**
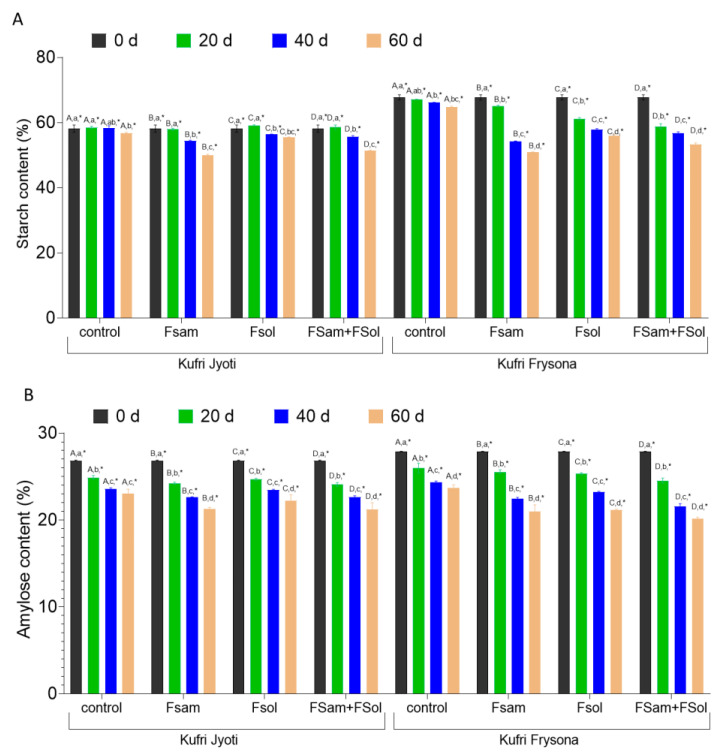
The effect of dry rot infection on (**A**) starch content (**B**) amylose content in cultivars Kufri Jyoti and Kufri Frysona; control, no pathogen infection; FSam, *Fusarium sambucinum*; FSol, *Fusarium solani*; FSam + FSol, *Fusarium sambucinum* + *Fusarium solani*. Capital letter = significance of inoculum (within one date/cultivar combo); Lower case letter = significance of date (within one inoculum/cultivar combo); * = significance between cultivars (within one inoculum/date combo).

In this study, the average starch content in freshly harvested tubers was recorded as up to 60.29% in Kufri Jyoti and 67.81% in Kufri Frysona. The starch content was observed to deteriorate significantly (Supplementary Table S6, *p* < 0.05) during storage of potato tubers for two months (estimated after 0, 20, 40, and 60 days). Our results show that the infection with the individual (either FSam or FSol) or combined (FSam + FSol) fungi caused a significant reduction in starch content when tubers were stored for 0, 20, 40, and 60 days of infection (Figure 5A, Supplementary Table S6). Irrespective of the cultivars, FSam-infected tubers were shown to have a more significant reduction (Table S5, *p* < 0.05) in starch content as compared to FSol-infected tubers. The starch content in FSam infected tubers of Kufri Jyoti and Kufri Frysona after 60 days of inoculation was 49.94% and 50.89%, respectively. Likewise, in FSol-infected tubers of Kufri Jyoti and Kufri Frysona, the average starch content was 55.5 and 60.01%, respectively, at the end of two months. Interestingly, the combination of FSam + FSol caused higher reduction in starch content after 60 days, and there was a decrease in starch content by 14.4% and 21.2% in Kufri Jyoti and Kufri Frysona, respectively. 

These results demonstrated that FSam not only caused a higher lesion index and rot volume but severely affected starch metabolism and, ultimately, caused a greater reduction in starch content as compared to FSol in both cultivars. Initially, the starch content of Kufri Jyoti tubers was not significantly reduced at 20 days post infection when compared to the control. Similarly, there was a significant reduction in starch content after 40 and 60 days of infection by FSol in Kufri Jyoti (Figure 5A). Nevertheless, the mixed infection (FSam + FSol) severely affected the starch content in tubers of Kufri Jyoti and Kufri Frysona. These findings signify that the temporal effect of FSam, FSol, and FSam + FSol show a prominent and significant reduction in starch content after 20, 40, and 60 d of infection (Figure 5).

Amylose, a lined chain of glucose molecules with an α (1,4) linkage, is vulnerable to hydrolysis by α-amylase. Our results showed that dry rot disease development with the infection of FSam, FSol, and FSam + FSol caused a substantial reduction in amylose content in Kufri Jyoti and Kufri Frysona potato cultivars (Figure 5B). The amylose content of uninoculated Kufri Jyoti and Kufri Frysona was 26.9% and 27.8%, respectively, at day 0, but declined to 23.06% and 23.7% by day 60 (Figure 5B). The infection with FSam in potato tubers of Kufri Jyoti and Kufri Frysona caused a severe reduction in amylose content to 21.3% and 20.9% after two months of infection as compared to control tubers. Similarly, the amylose content in FSol infected tubers of Kufri Jyoti and Kufri Frysona deteriorated to 22.2% and 21.1%, respectively, as compared to the control. Furthermore, the combination of fungal infection (FSam + FSol) in Kufri Jyoti and Kufri Frysona caused a significant decrease (*p* < 0.05) in amylose content to 21.2% and 20.1%, respectively. 

### 3.7. Dry Rot Infection Affects Glycemic Index (GI), Resistant Starch (RS), and Glycemic Load (GL) of Potato Tubers

**Figure 6:**
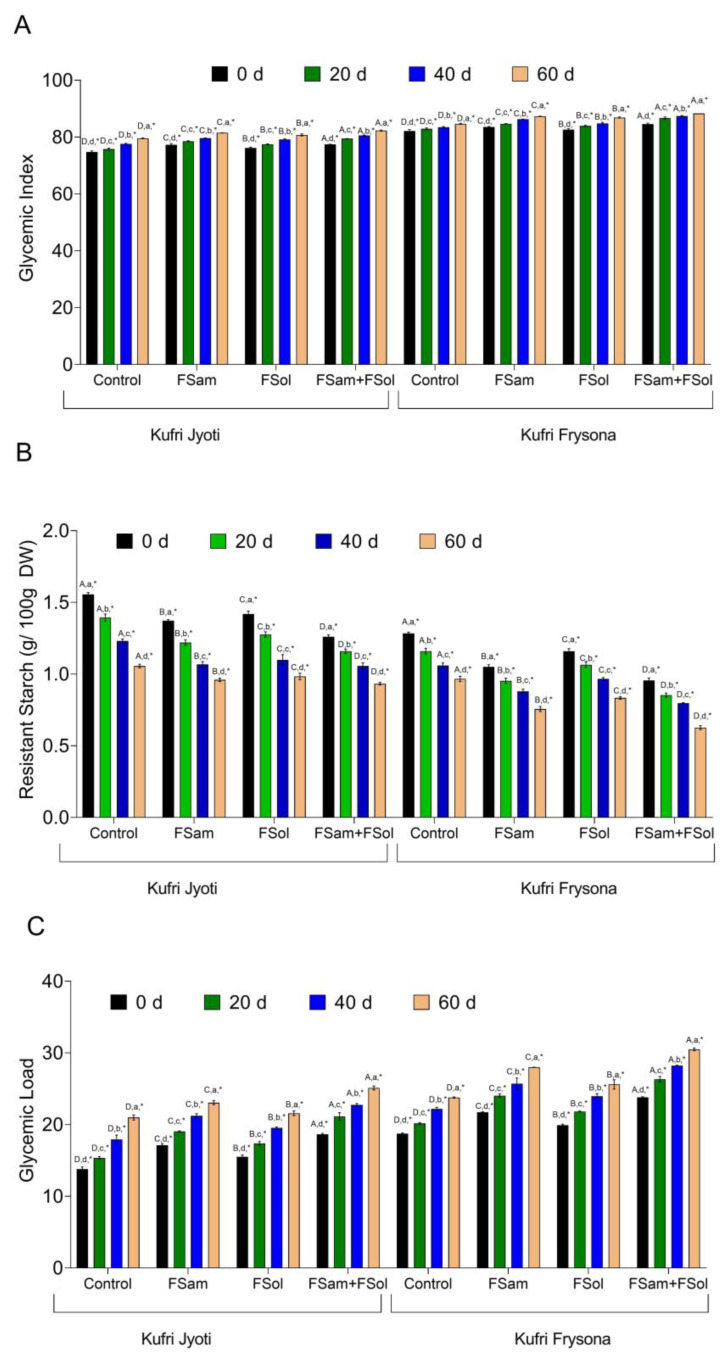
The effect of dry rot infection on (**A**) glycemic index, (**B**) resistant starch, and (**C**) glycemic load of cultivars Kufri Jyoti and Kufri Frysona; control, no pathogen infection; FSam, *Fusarium sambucinum*; Fsol, *Fusarium solani*; Fsam + Fsol, *Fusarium sambucinum* + *Fusarium solani.* Capital letter = significance of inoculum (within one date/cultivar combo); Lower case letter = significance of date (within one inoculum/cultivar combo); * = significance between cultivars (within one inoculum/date combo).

The effect of pathogen infection on GI, RS, and GL of starchy crops has gained limited attention, and studies are elusive on this topic. In this study, our ultimate aim was to determine glycemic response traits such as GI, RS, and GL parameters, which are essential from the perspective of human health. The results revealed that the GI of Kufri Frysona and Kufri Jyoti was significantly (Table S7, *p* < 0.01) elevated in rot-affected tubers as compared to the control group treatments (Figure 6A). In control tubers at the time of storage, the GI value for Kufri Jyoti was 74.6, which significantly increased to 81.5, 80.7, and 82.3, respectively in FSam, FSol, and FSam + FSol inoculated tubers. Likewise, the GI value of 82.1 of Kufri Frysona (control tubers) increased under the dry rot infection to 87.3, 86.8, and 88.1, respectively for FSam, FSol, and FSam + FSol inoculated tubers. The percent increase in GI of tubers was higher in Kufri Jyoti than in Kufri Frysona. Likewise, the FSam infected tubers had proportionately higher GI than did FSol infected tubers. However, the mixed infection caused a much higher GI increase than did the rest of the treatments. 

Another integral component of high nutritional importance is the resistant starch of potato tubers. The starch is left undigested in the small intestine due to its digestion-resistant nature and makes its way as is to the large intestine for fermentation. We observed that the content of resistant starch was significantly affected in dry rot infected tubers (Figure 6B). The RS content varied significantly (Table S7, *p* < 0.01) due to FSam, FSol, and FSam + FSol treatment on both varieties. The initial RS content of Kufri Jyoti and Kufri Frysona in the control was reported as 1.5% and 1.26%. However, RS in the tuber of Kufri Jyoti was found to be reduced to 0.94% and 0.99% in FSam and FSol-infected tubers, respectively. The RS content was 0.91% in the combined infection. A similar pattern of significant reduction in RS was observed in Kufri Frysona for all treatment combinations. 

In this study, we reported for the first time the effect of tuber-borne infection on the GL of potato. In control tubers of Kufri Jyoti and Kufri Frysona, the GL was 13.8 and 18.7, respectively (Figure 6C). After 60 days of *Fusarium* infection (FSam), the GL of tubers of Kufri Jyoti and Kufri Frysona increased to 23.07 and 28.02, respectively. Similarly, in FSol-infected tubers of Kufri Jyoti and Kufri Frysona, the GL increased to 21.5 and 25.6, respectively.

### 3.8. Pearson Correlation Analysis between the Parameters of Dry Rot Infection and Glycemic Response Traits

The Pearson correlation analysis revealed that the lesion diameter, lesion depth, and rot volume had a negative correlation of −0.79, −0.78, and −0.64, respectively (Table 1). Similarly, the amylose content was also negatively correlated with the infection due to both fungi. The quality aspect of the potato tuber is highly dependent on the starch grain and amylose content of the potato tuber. Moreover, our results suggest that fungal infection also affects glycemic response traits such as GI, GL, and RS (Table 1). 

## 4. Discussion

Dry rot disease in potato is caused by more than 13 Fusarium species worldwide [6]. In major parts of Europe, China, North America, and Asia, Fusarium sambucinum, Fusarium solani, and Fusarium oxysporum are recognized as the most aggressive fungi responsible for this disease. Previously, these two species, namely Fusarium sambucinum and Fusarium solani, have been extensively reported to cause dry rot in potato tubers [10,38]. We observed a higher level of disease tolerance in Kufri Frysona as compared to Kufri Jyoti. It will be more appropriate to cultivate Kufri Frysona as a processing variety in dry rot prone areas. Our findings are in agreement with previous reports that have highlighted the higher susceptibility of table purpose cultivars to dry rot infection as compared to processing varieties that had a high dry matter content [39]. The findings in our experiments also support the previously established facts that the lesion depth and fungus penetration depend on the cultivar types, the species of fungus involved, and the duration of storage post-inoculation [5,38,39]. Mejdoub Trabelsi et al. have shown a significantly higher depth of penetration in inoculated tubers with combinations of Fusarium sambucinum with Fusarium oxysporum when compared to individual Fusarium species inoculation [38]. Our findings are in agreement with results of Mejdoub Trabelsi et al., as they discovered that mixtures of Fusarium sambucinum with Fusarium oxysporum caused enhanced rot volume in four potato varieties, namely Spunta, Oceanea, Mondial, and Nikola [38]. Even in other food commodities and crops, similar findings exist where the disease index varies based on the species of fungus involved in causing infection. Higher disease severity due to infection of one or more fungal species was also reported in diseases of maize, wheat alfalfa, and soybean [40,41,42]. The foot and crown rot disease of wheat was reported to occur in a more severe form under the presence of Fusarium graminearum, Fusarium culmorum, Fusarium poae, and Fusarium sporotrichioides [29,43,44]. Starch is an integral component of carbohydrate forms available in the potato, mainly required to provide metabolic energy that empowers the human or animal body to perform its activities [27,45,46,47,48]. It has been previously reported that biotic stress such as virus infection was shown to have a detrimental effect on starch quality-related parameters. Lal et al. reported that the apical leaf curl disease infection in Kufri Pukhraj potatoes caused a severe reduction in starch content during storage [44]. Additionally, it was previously recorded that Fusarium sambucinum and Fusarium oxysporum infection led to a substantial reduction in starch content in Kufri Pukhraj and Kufri Chipsona varieties [16]. In the sweet potato, similar results were documented where leaf curl virus infection significantly deteriorated the starch content in tubers by 9.3% during the active growing phase of the crop [48]. The mixture of FSam and FSol resulted in a substantial decline (p < 0.05) in amylose content irrespective of inoculated cultivar (Kufri Jyoti and Kufri Frysona). Previous reports suggest that the amylose chain might be degraded by α-amylase, which is produced by fungus alone (Fsam or FO) or in a mixture (Fsam + FO) [16]. The findings of this experiments are in consensus with previous reports of correlation of amylose reduction under fungal infection in crop plants [16]. Even in potato tubers of cultivars Kufri Pukhraj and Kufri Bahar, the viral infection had significantly deteriorated the amylose content as per the previous report [44].

Nevertheless, the effect of major physiological adversities, drought stress, low light, and phytic acid-mediated effects on GI and RS of rice grains was previously studied. The high GI might be attributed to the reduction in amylose content in the tubers [22,29,46]. It is well established that GI is negatively correlated with the amylose content in rice, potato, and sweet potato crops [20,28,46,47,48]. Previous reports also highlight that GI was substantially influenced by the structure and composition of starch [22,28,46]. Our findings also support these established facts that GI was affected, as the composition of starch changed under Fusarium infection. There are existing reports of RS reduction in starch crops under the influence of drought stress and viral infections [29,44].

Several studies document the adverse influence of high GI foods on human health [22,27,29,45]. Our findings are in consensus with previous reports of increased GL due to pathogen infection [44]. Our findings indicated that under the influence of fungal infection, the tubers gained high GI and GL, which are not suitable for consumers from the perspective of good health. The losses due to dry rot infection are not only quantitative, in terms of increased rot volume, but qualitative too, which is a matter of concern in the potato production system.

The rot volume, lesion depth, and lesion diameter were progressive, and there was an increase in GI and GL. This might be due to the hydrolysis of starch due to fungal infection, which ultimately leads to the enhancement of free glucose and sugar. The free glucose and sugar ultimately affect the GI and GL of potato tubers and thereby enhance these traits. Moreover, the results revealed that higher infection showed lower RS content. The correlation between the starch traits and glycemic response traits is concordant with the previous studies. These results suggest that infection in the potato tuber affects not only the quality aspect of potato but also the nutritional and health characteristics.

## 5. Conclusions

Potato dry rot has emerged as a major soil and tuber-borne storage disease that hampers the economic value of the potato after harvesting. Our study showed a deteriorating quantitative and qualitative effect of this disease on table and processing cultivars of potato. However, Kufri Frysona was more tolerant to dry rot infection, which suggests its suitability for cultivation in dry rot-prone areas. Fusarium sambucinum and Fusarium solani, which are very common causal species of dry rot, significantly enhanced the disease severity. Over the storage duration of two months, the lesion diameter, fungus penetration, and rot volume increased significantly in a species-specific and cultivar-specific manner. The fungal infection severely hampered the starch content and amylose content in table and processing varieties. Additionally, the GI and GL of potato tubers were substantially increased in the infected tubers as compared to the control. The RS, which is beneficial for gut health, was decreased under Fusarium infection. The potato already falls into the category of high GI food crop and, as a result of infection, the proportionate increase in GI and GL and the substantial reduction of RS are concerning from the perspective of consumer health in general and diabetes in particular. 

## Figures and Tables

**Table 1:** Pearson correlation coefficient of the different parameters in Kufri Jyoti and Kufri Frysona that were infected with control (no fungal infection), FSam, FSol, and FSam + Fsol for 0, 20, 40, and 60 d of infection.

Parameters	Lesion Diameter	Lesion Depth	Rot Volume	Starch	Amylose	Glycemic Index	Glycemic Load	Resistant Starch
Lesion Diameter	1.00							
Lesion Depth	0.96	1.00						
Rot Volume	0.82	0.87	1.00					
Starch	−0.79	−0.78	−0.64	1.00				
Amylose	−0.92	−0.88	−0.64	0.82	1.00			
Glycemic Index	0.30	0.31	0.15	−0.03	−0.41	1.00		
Glycemic Load	0.56	0.59	0.40	−0.30	−0.63	0.92	1.00	
Resistant Starch	−0.60	−0.59	−0.41	0.29	0.65	−0.91	−0.96	1.00

Abbreviations: FSam, *Fusarium sambucinum*; FSol, *Fusarium solani*; FSam + FSol, *Fusarium sambucinum* + *Fusarium solani*.

## Data Availability

All data generated or analyzed during this study are included in this published article and its supplementary information files.

## Supplementary Materials

The following supporting information can be downloaded at: https://www.mdpi.com/article/10.3390/jof9040466/s1, Table S1: Tukey multiple comparison test for lesion diameter of Kufri Jyoti when infected with control (water agar), Fusarium sambucinum, Fusarium solani, and Fusarium sambucinum + Fusarium solani and incubated for 0 d, 20 d, 40 d, and 60 days; Table S2: Tukey multiple comparison test for lesion diameter of Kufri Frysona when infected with control (water agar), Fusarium sambucinum, Fusarium solani, and Fusarium sambucinum + Fusarium solani and incubated for 0 d, 20 d, 40 d, and 60 days; Table S3: Tukey multiple comparison test for lesion depth of Kufri Jyoti when infected with control (water agar), Fusarium sambucinum, Fusarium solani, and Fusarium sambucinum + Fusarium solani and incubated for 0 d, 20 d, 40 d, and 60 days; Table S4: Tukey multiple comparison test for lesion depth of Kufri Frysona when infected with control (water agar), Fusarium sambucinum, Fusarium solani, and Fusarium sambucinum + Fusarium solani and incubated for 0 d, 20 d, 40 d, and 60 days; Table S5: Bonferroni’s multiple comparisons test for rot volume of Kufri Jyoti and Kufri Frysona when infected with control (water agar), Fusarium sambucinum, Fusarium solani, and Fusarium sambucinum + Fusarium solani and incubated for 0 d, 20 d, 40 d, and 60 days; Table S6: Tukey multiple comparison test for starch content of Kufri Jyoti and Kufri Frysona when infected with control (water agar), Fusarium sambucinum, Fusarium solani, and Fusarium sambucinum + Fusarium solani and incubated for 0 d, 20 d, 40 d, and 60 days; Table S7: Tukey multiple comparison test for glycemic index of Kufri Jyoti and Kufri Frysona when infected with control (water agar), Fusarium sambucinum, Fusarium solani, and Fusarium sambucinum + Fusarium solani and incubated for 0 d, 20 d, 40 d, and 60 days.
