# Supplementary material for: Impact of Fusarium Infection on Potato Quality, Starch Digestibility, In Vitro Glycemic Response, and Resistant Starch Content
Source: J Fungi (Basel). 2023 Apr 12;9(4):466. doi: 10.3390/jof9040466 (PMC10144655; doi:10.3390/jof9040466)
Supplement: Supplementary file 1 [file jof-09-00466-s001.zip › jof-2245514-supplementary.pdf]

Supplementary tables:

Table S1: Tukey multiple comparison test for lesion diameter of Kufri Jyoti when infected with control (water agar), *Fusarium Sambucinum*, *Fusarium solani* and *Fusarium Sambucinum* + *Fusarium solani* and incubated for 0 d, 20 d, 40 d and 60 days

Tukey's multiple comparisons test	Mean Diff.	95.00% CI of diff.	Significant?	Summary	Adjusted P Value
Control:0 d vs. Control:20 d	-1.1E-14	-2.498 to 2.498	No	ns	>0.9999
Control:0 d vs. Control:40 d	-1.1E-14	-2.498 to 2.498	No	ns	>0.9999
Control:0 d vs. Control:60 d	-1.1E-14	-2.498 to 2.498	No	ns	>0.9999
Control:0 d vs. FSam:0 d	-3.6E-15	-2.498 to 2.498	No	ns	>0.9999
Control:0 d vs. FSam:20 d	-13.73	-16.23 to -11.24	Yes	****	<0.0001
Control:0 d vs. FSam:40 d	-25.5	-28.00 to -23.00	Yes	****	<0.0001
Control:0 d vs. FSam:60 d	-39.6	-42.10 to -37.10	Yes	****	<0.0001
Control:0 d vs. FSol:0 d	-1.1E-14	-2.498 to 2.498	No	ns	>0.9999
Control:0 d vs. FSol:20 d	-11.87	-14.36 to -9.369	Yes	****	<0.0001
Control:0 d vs. FSol:40 d	-20.73	-23.23 to -18.24	Yes	****	<0.0001
Control:0 d vs. FSol:60 d	-27.57	-30.06 to -25.07	Yes	****	<0.0001
Control:0 d vs. FSam+FSol:0 d	-1.1E-14	-2.498 to 2.498	No	ns	>0.9999
Control:0 d vs. FSam+FSol:20 d	-15.07	-17.56 to -12.57	Yes	****	<0.0001
Control:0 d vs. FSam+FSol:40 d	-27.7	-30.20 to -25.20	Yes	****	<0.0001
Control:0 d vs. FSam+FSol:60 d	-40.83	-43.33 to -38.34	Yes	****	<0.0001
Control:20 d vs. Control:40 d	0	-2.498 to 2.498	No	ns	>0.9999
Control:20 d vs. Control:60 d	0	-2.498 to 2.498	No	ns	>0.9999
Control:20 d vs. FSam:0 d	7.11E-15	-2.498 to 2.498	No	ns	>0.9999
Control:20 d vs. FSam:20 d	-13.73	-16.23 to -11.24	Yes	****	<0.0001
Control:20 d vs. FSam:40 d	-25.5	-28.00 to -23.00	Yes	****	<0.0001
Control:20 d vs. FSam:60 d	-39.6	-42.10 to -37.10	Yes	****	<0.0001
Control:20 d vs. FSol:0 d	0	-2.498 to 2.498	No	ns	>0.9999

Control:20 d vs. FSol:20 d	-11.87	-14.36 to -9.369	Yes	****	<0.0001
Control:20 d vs. FSol:40 d	-20.73	-23.23 to -18.24	Yes	****	<0.0001
Control:20 d vs. FSol:60 d	-27.57	-30.06 to -25.07	Yes	****	<0.0001
Control:20 d vs. FSam+FSol:0 d	0	-2.498 to 2.498	No	ns	>0.9999
Control:20 d vs. FSam+FSol:20 d	-15.07	-17.56 to -12.57	Yes	****	<0.0001
Control:20 d vs. FSam+FSol:40 d	-27.7	-30.20 to -25.20	Yes	****	<0.0001
Control:20 d vs. FSam+FSol:60 d	-40.83	-43.33 to -38.34	Yes	****	<0.0001
Control:40 d vs. Control:60 d	0	-2.498 to 2.498	No	ns	>0.9999
Control:40 d vs. FSam:0 d	7.11E-15	-2.498 to 2.498	No	ns	>0.9999
Control:40 d vs. FSam:20 d	-13.73	-16.23 to -11.24	Yes	****	<0.0001
Control:40 d vs. FSam:40 d	-25.5	-28.00 to -23.00	Yes	****	<0.0001
Control:40 d vs. FSam:60 d	-39.6	-42.10 to -37.10	Yes	****	<0.0001
Control:40 d vs. FSol:0 d	0	-2.498 to 2.498	No	ns	>0.9999
Control:40 d vs. FSol:20 d	-11.87	-14.36 to -9.369	Yes	****	<0.0001
Control:40 d vs. FSol:40 d	-20.73	-23.23 to -18.24	Yes	****	<0.0001
Control:40 d vs. FSol:60 d	-27.57	-30.06 to -25.07	Yes	****	<0.0001
Control:40 d vs. FSam+FSol:0 d	0	-2.498 to 2.498	No	ns	>0.9999
Control:40 d vs. FSam+FSol:20 d	-15.07	-17.56 to -12.57	Yes	****	<0.0001
Control:40 d vs. FSam+FSol:40 d	-27.7	-30.20 to -25.20	Yes	****	<0.0001
Control:40 d vs. FSam+FSol:60 d	-40.83	-43.33 to -38.34	Yes	****	<0.0001
Control:60 d vs. FSam:0 d	7.11E-15	-2.498 to 2.498	No	ns	>0.9999
Control:60 d vs. FSam:20 d	-13.73	-16.23 to -11.24	Yes	****	<0.0001
Control:60 d vs. FSam:40 d	-25.5	-28.00 to -23.00	Yes	****	<0.0001
Control:60 d vs. FSam:60 d	-39.6	-42.10 to -37.10	Yes	****	<0.0001
Control:60 d vs. FSol:0 d	0	-2.498 to 2.498	No	ns	>0.9999
Control:60 d vs. FSol:20 d	-11.87	-14.36 to -9.369	Yes	****	<0.0001
Control:60 d vs. FSol:40 d	-20.73	-23.23 to -18.24	Yes	****	<0.0001

Control:60 d vs. FSol:60 d	-27.57	-30.06 to -25.07	Yes	****	<0.0001
Control:60 d vs. FSam+FSol:0 d	0	-2.498 to 2.498	No	ns	>0.9999
Control:60 d vs. FSam+FSol:20 d	-15.07	-17.56 to -12.57	Yes	****	<0.0001
Control:60 d vs. FSam+FSol:40 d	-27.7	-30.20 to -25.20	Yes	****	<0.0001
Control:60 d vs. FSam+FSol:60 d	-40.83	-43.33 to -38.34	Yes	****	<0.0001
FSam:0 d vs. FSam:20 d	-13.73	-16.23 to -11.24	Yes	****	<0.0001
FSam:0 d vs. FSam:40 d	-25.5	-28.00 to -23.00	Yes	****	<0.0001
FSam:0 d vs. FSam:60 d	-39.6	-42.10 to -37.10	Yes	****	<0.0001
FSam:0 d vs. FSol:0 d	-7.1E-15	-2.498 to 2.498	No	ns	>0.9999
FSam:0 d vs. FSol:20 d	-11.87	-14.36 to -9.369	Yes	****	<0.0001
FSam:0 d vs. FSol:40 d	-20.73	-23.23 to -18.24	Yes	****	<0.0001
FSam:0 d vs. FSol:60 d	-27.57	-30.06 to -25.07	Yes	****	<0.0001
FSam:0 d vs. FSam+FSol:0 d	-7.1E-15	-2.498 to 2.498	No	ns	>0.9999
FSam:0 d vs. FSam+FSol:20 d	-15.07	-17.56 to -12.57	Yes	****	<0.0001
FSam:0 d vs. FSam+FSol:40 d	-27.7	-30.20 to -25.20	Yes	****	<0.0001
FSam:0 d vs. FSam+FSol:60 d	-40.83	-43.33 to -38.34	Yes	****	<0.0001
FSam:20 d vs. FSam:40 d	-11.77	-14.26 to -9.269	Yes	****	<0.0001
FSam:20 d vs. FSam:60 d	-25.87	-28.36 to -23.37	Yes	****	<0.0001
FSam:20 d vs. FSol:0 d	13.73	11.24 to 16.23	Yes	****	<0.0001
FSam:20 d vs. FSol:20 d	1.867	-0.6311 to 4.364	No	ns	0.3333
FSam:20 d vs. FSol:40 d	-7	-9.498 to -4.502	Yes	****	<0.0001
FSam:20 d vs. FSol:60 d	-13.83	-16.33 to -11.34	Yes	****	<0.0001
FSam:20 d vs. FSam+FSol:0 d	13.73	11.24 to 16.23	Yes	****	<0.0001
FSam:20 d vs. FSam+FSol:20 d	-1.333	-3.831 to 1.164	No	ns	0.8189
FSam:20 d vs. FSam+FSol:40 d	-13.97	-16.46 to -11.47	Yes	****	<0.0001
FSam:20 d vs. FSam+FSol:60 d	-27.1	-29.60 to -24.60	Yes	****	<0.0001
FSam:40 d vs. FSam:60 d	-14.1	-16.60 to -11.60	Yes	****	<0.0001

FSam:40 d vs. FSol:0 d	25.5	23.00 to 28.00	Yes	****	<0.0001
FSam:40 d vs. FSol:20 d	13.63	11.14 to 16.13	Yes	****	<0.0001
FSam:40 d vs. FSol:40 d	4.767	2.269 to 7.264	Yes	****	<0.0001
FSam:40 d vs. FSol:60 d	-2.067	-4.564 to 0.4311	No	ns	0.1975
FSam:40 d vs. FSam+FSol:0 d	25.5	23.00 to 28.00	Yes	****	<0.0001
FSam:40 d vs. FSam+FSol:20 d	10.43	7.936 to 12.93	Yes	****	<0.0001
FSam:40 d vs. FSam+FSol:40 d	-2.2	-4.698 to 0.2978	No	ns	0.1333
FSam:40 d vs. FSam+FSol:60 d	-15.33	-17.83 to -12.84	Yes	****	<0.0001
FSam:60 d vs. FSol:0 d	39.6	37.10 to 42.10	Yes	****	<0.0001
FSam:60 d vs. FSol:20 d	27.73	25.24 to 30.23	Yes	****	<0.0001
FSam:60 d vs. FSol:40 d	18.87	16.37 to 21.36	Yes	****	<0.0001
FSam:60 d vs. FSol:60 d	12.03	9.536 to 14.53	Yes	****	<0.0001
FSam:60 d vs. FSam+FSol:0 d	39.6	37.10 to 42.10	Yes	****	<0.0001
FSam:60 d vs. FSam+FSol:20 d	24.53	22.04 to 27.03	Yes	****	<0.0001
FSam:60 d vs. FSam+FSol:40 d	11.9	9.402 to 14.40	Yes	****	<0.0001
FSam:60 d vs. FSam+FSol:60 d	-1.233	-3.731 to 1.264	No	ns	0.8869
FSol:0 d vs. FSol:20 d	-11.87	-14.36 to -9.369	Yes	****	<0.0001
FSol:0 d vs. FSol:40 d	-20.73	-23.23 to -18.24	Yes	****	<0.0001
FSol:0 d vs. FSol:60 d	-27.57	-30.06 to -25.07	Yes	****	<0.0001
FSol:0 d vs. FSam+FSol:0 d	0	-2.498 to 2.498	No	ns	>0.9999
FSol:0 d vs. FSam+FSol:20 d	-15.07	-17.56 to -12.57	Yes	****	<0.0001
FSol:0 d vs. FSam+FSol:40 d	-27.7	-30.20 to -25.20	Yes	****	<0.0001
FSol:0 d vs. FSam+FSol:60 d	-40.83	-43.33 to -38.34	Yes	****	<0.0001
FSol:20 d vs. FSol:40 d	-8.867	-11.36 to -6.369	Yes	****	<0.0001
FSol:20 d vs. FSol:60 d	-15.7	-18.20 to -13.20	Yes	****	<0.0001
FSol:20 d vs. FSam+FSol:0 d	11.87	9.369 to 14.36	Yes	****	<0.0001
FSol:20 d vs. FSam+FSol:20 d	-3.2	-5.698 to -0.7022	Yes	**	0.0035

FSol:20 d vs. FSam+FSol:40 d	-15.83	-18.33 to -13.34	Yes	****	<0.0001
FSol:20 d vs. FSam+FSol:60 d	-28.97	-31.46 to -26.47	Yes	****	<0.0001
FSol:40 d vs. FSol:60 d	-6.833	-9.331 to -4.336	Yes	****	<0.0001
FSol:40 d vs. FSam+FSol:0 d	20.73	18.24 to 23.23	Yes	****	<0.0001
FSol:40 d vs. FSam+FSol:20 d	5.667	3.169 to 8.164	Yes	****	<0.0001
FSol:40 d vs. FSam+FSol:40 d	-6.967	-9.464 to -4.469	Yes	****	<0.0001
FSol:40 d vs. FSam+FSol:60 d	-20.1	-22.60 to -17.60	Yes	****	<0.0001
FSol:60 d vs. FSam+FSol:0 d	27.57	25.07 to 30.06	Yes	****	<0.0001
FSol:60 d vs. FSam+FSol:20 d	12.5	10.00 to 15.00	Yes	****	<0.0001
FSol:60 d vs. FSam+FSol:40 d	-0.1333	-2.631 to 2.364	No	ns	>0.9999
FSol:60 d vs. FSam+FSol:60 d	-13.27	-15.76 to -10.77	Yes	****	<0.0001
FSam+FSol:0 d vs. FSam+FSol:20 d	-15.07	-17.56 to -12.57	Yes	****	<0.0001
FSam+FSol:0 d vs. FSam+FSol:40 d	-27.7	-30.20 to -25.20	Yes	****	<0.0001
FSam+FSol:0 d vs. FSam+FSol:60 d	-40.83	-43.33 to -38.34	Yes	****	<0.0001
FSam+FSol:20 d vs. FSam+FSol:40 d	-12.63	-15.13 to -10.14	Yes	****	<0.0001
FSam+FSol:20 d vs. FSam+FSol:60 d	-25.77	-28.26 to -23.27	Yes	****	<0.0001
FSam+FSol:40 d vs. FSam+FSol:60 d	-13.13	-15.63 to -10.64	Yes	****	<0.0001

Table S2: Tukey multiple comparison test for lesion diameter of Kufri Frysona when infected with control (water agar), *Fusarium Sambucinum*, *Fusarium solani* and *Fusarium Sambucinum* + *Fusarium solani* and incubated for 0 d, 20 d, 40 d and 60 days

Tukey's multiple comparisons test	Mean Diff.	95.00% CI of diff.	Significant ?	Summary	Adjusted P Value
Control:0 d vs. Control:20 d	7.11E-15	-2.250 to 2.250	No	ns	>0.9999
Control:0 d vs. Control:40 d	0	-2.250 to 2.250	No	ns	>0.9999
Control:0 d vs. Control:60 d	0	-2.250 to 2.250	No	ns	>0.9999
Control:0 d vs. FSam:0 d	0	-2.250 to 2.250	No	ns	>0.9999
Control:0 d vs. FSam:20 d	-12.27	-14.52 to -10.02	Yes	****	<0.0001

Control:0 d vs. FSam:40 d	-20	-22.25 to -17.75	Yes	****	<0.0001
Control:0 d vs. FSam:60 d	-24.87	-27.12 to -22.62	Yes	****	<0.0001
Control:0 d vs. FSol:0 d	-5.3E-15	-2.250 to 2.250	No	ns	>0.9999
Control:0 d vs. FSol:20 d	-10.23	-12.48 to -7.983	Yes	****	<0.0001
Control:0 d vs. FSol:40 d	-16.37	-18.62 to -14.12	Yes	****	<0.0001
Control:0 d vs. FSol:60 d	-25.03	-27.28 to -22.78	Yes	****	<0.0001
Control:0 d vs. FSam+FSol:0 d	0	-2.250 to 2.250	No	ns	>0.9999
Control:0 d vs. FSam+FSol:20 d	-12.97	-15.22 to -10.72	Yes	****	<0.0001
Control:0 d vs. FSam+FSol:40 d	-21.6	-23.85 to -19.35	Yes	****	<0.0001
Control:0 d vs. FSam+FSol:60 d	-33.3	-35.55 to -31.05	Yes	****	<0.0001
Control:20 d vs. Control:40 d	-7.1E-15	-2.250 to 2.250	No	ns	>0.9999
Control:20 d vs. Control:60 d	-7.1E-15	-2.250 to 2.250	No	ns	>0.9999
Control:20 d vs. FSam:0 d	-7.1E-15	-2.250 to 2.250	No	ns	>0.9999
Control:20 d vs. FSam:20 d	-12.27	-14.52 to -10.02	Yes	****	<0.0001
Control:20 d vs. FSam:40 d	-20	-22.25 to -17.75	Yes	****	<0.0001
Control:20 d vs. FSam:60 d	-24.87	-27.12 to -22.62	Yes	****	<0.0001
Control:20 d vs. FSol:0 d	-1.2E-14	-2.250 to 2.250	No	ns	>0.9999
Control:20 d vs. FSol:20 d	-10.23	-12.48 to -7.983	Yes	****	<0.0001
Control:20 d vs. FSol:40 d	-16.37	-18.62 to -14.12	Yes	****	<0.0001
Control:20 d vs. FSol:60 d	-25.03	-27.28 to -22.78	Yes	****	<0.0001
Control:20 d vs. FSam+FSol:0 d	-7.1E-15	-2.250 to 2.250	No	ns	>0.9999
Control:20 d vs. FSam+FSol:20 d	-12.97	-15.22 to -10.72	Yes	****	<0.0001
Control:20 d vs. FSam+FSol:40 d	-21.6	-23.85 to -19.35	Yes	****	<0.0001
Control:20 d vs. FSam+FSol:60 d	-33.3	-35.55 to -31.05	Yes	****	<0.0001
Control:40 d vs. Control:60 d	0	-2.250 to 2.250	No	ns	>0.9999
Control:40 d vs. FSam:0 d	0	-2.250 to 2.250	No	ns	>0.9999
Control:40 d vs. FSam:20 d	-12.27	-14.52 to -10.02	Yes	****	<0.0001

Control:40 d vs. FSam:40 d	-20	-22.25 to -17.75	Yes	****	<0.0001
Control:40 d vs. FSam:60 d	-24.87	-27.12 to -22.62	Yes	****	<0.0001
Control:40 d vs. FSol:0 d	-5.3E-15	-2.250 to 2.250	No	ns	>0.9999
Control:40 d vs. FSol:20 d	-10.23	-12.48 to -7.983	Yes	****	<0.0001
Control:40 d vs. FSol:40 d	-16.37	-18.62 to -14.12	Yes	****	<0.0001
Control:40 d vs. FSol:60 d	-25.03	-27.28 to -22.78	Yes	****	<0.0001
Control:40 d vs. FSam+FSol:0 d	0	-2.250 to 2.250	No	ns	>0.9999
Control:40 d vs. FSam+FSol:20 d	-12.97	-15.22 to -10.72	Yes	****	<0.0001
Control:40 d vs. FSam+FSol:40 d	-21.6	-23.85 to -19.35	Yes	****	<0.0001
Control:40 d vs. FSam+FSol:60 d	-33.3	-35.55 to -31.05	Yes	****	<0.0001
Control:60 d vs. FSam:0 d	0	-2.250 to 2.250	No	ns	>0.9999
Control:60 d vs. FSam:20 d	-12.27	-14.52 to -10.02	Yes	****	<0.0001
Control:60 d vs. FSam:40 d	-20	-22.25 to -17.75	Yes	****	<0.0001
Control:60 d vs. FSam:60 d	-24.87	-27.12 to -22.62	Yes	****	<0.0001
Control:60 d vs. FSol:0 d	-5.3E-15	-2.250 to 2.250	No	ns	>0.9999
Control:60 d vs. FSol:20 d	-10.23	-12.48 to -7.983	Yes	****	<0.0001
Control:60 d vs. FSol:40 d	-16.37	-18.62 to -14.12	Yes	****	<0.0001
Control:60 d vs. FSol:60 d	-25.03	-27.28 to -22.78	Yes	****	<0.0001
Control:60 d vs. FSam+FSol:0 d	0	-2.250 to 2.250	No	ns	>0.9999
Control:60 d vs. FSam+FSol:20 d	-12.97	-15.22 to -10.72	Yes	****	<0.0001
Control:60 d vs. FSam+FSol:40 d	-21.6	-23.85 to -19.35	Yes	****	<0.0001
Control:60 d vs. FSam+FSol:60 d	-33.3	-35.55 to -31.05	Yes	****	<0.0001
FSam:0 d vs. FSam:20 d	-12.27	-14.52 to -10.02	Yes	****	<0.0001
FSam:0 d vs. FSam:40 d	-20	-22.25 to -17.75	Yes	****	<0.0001
FSam:0 d vs. FSam:60 d	-24.87	-27.12 to -22.62	Yes	****	<0.0001
FSam:0 d vs. FSol:0 d	-5.3E-15	-2.250 to 2.250	No	ns	>0.9999
FSam:0 d vs. FSol:20 d	-10.23	-12.48 to -7.983	Yes	****	<0.0001

FSam:0 d vs. FSol:40 d	-16.37	-18.62 to - 14.12	Yes	****	<0.0001
FSam:0 d vs. FSol:60 d	-25.03	-27.28 to - 22.78	Yes	****	<0.0001
FSam:0 d vs. FSam+FSol:0 d	0	-2.250 to 2.250	No	ns	>0.9999
FSam:0 d vs. FSam+FSol:20 d	-12.97	-15.22 to - 10.72	Yes	****	<0.0001
FSam:0 d vs. FSam+FSol:40 d	-21.6	-23.85 to - 19.35	Yes	****	<0.0001
FSam:0 d vs. FSam+FSol:60 d	-33.3	-35.55 to - 31.05	Yes	****	<0.0001
FSam:20 d vs. FSam:40 d	-7.733	-9.984 to - 5.483	Yes	****	<0.0001
FSam:20 d vs. FSam:60 d	-12.6	-14.85 to - 10.35	Yes	****	<0.0001
FSam:20 d vs. FSol:0 d	12.27	10.02 to 14.52	Yes	****	<0.0001
FSam:20 d vs. FSol:20 d	2.033	-0.2171 to 4.284	No	ns	0.1117
FSam:20 d vs. FSol:40 d	-4.1	-6.350 to - 1.850	Yes	****	<0.0001
FSam:20 d vs. FSol:60 d	-12.77	-15.02 to - 10.52	Yes	****	<0.0001
FSam:20 d vs. FSam+FSol:0 d	12.27	10.02 to 14.52	Yes	****	<0.0001
FSam:20 d vs. FSam+FSol:20 d	-0.7	-2.950 to 1.550	No	ns	0.9979
FSam:20 d vs. FSam+FSol:40 d	-9.333	-11.58 to - 7.083	Yes	****	<0.0001
FSam:20 d vs. FSam+FSol:60 d	-21.03	-23.28 to - 18.78	Yes	****	<0.0001
FSam:40 d vs. FSam:60 d	-4.867	-7.117 to - 2.616	Yes	****	<0.0001
FSam:40 d vs. FSol:0 d	20	17.75 to 22.25	Yes	****	<0.0001
FSam:40 d vs. FSol:20 d	9.767	7.516 to 12.02	Yes	****	<0.0001
FSam:40 d vs. FSol:40 d	3.633	1.383 to 5.884	Yes	***	0.0001
FSam:40 d vs. FSol:60 d	-5.033	-7.284 to - 2.783	Yes	****	<0.0001
FSam:40 d vs. FSam+FSol:0 d	20	17.75 to 22.25	Yes	****	<0.0001
FSam:40 d vs. FSam+FSol:20 d	7.033	4.783 to 9.284	Yes	****	<0.0001
FSam:40 d vs. FSam+FSol:40 d	-1.6	-3.850 to 0.6505	No	ns	0.4101
FSam:40 d vs. FSam+FSol:60 d	-13.3	-15.55 to - 11.05	Yes	****	<0.0001
FSam:60 d vs. FSol:0 d	24.87	22.62 to 27.12	Yes	****	<0.0001
FSam:60 d vs. FSol:20 d	14.63	12.38 to 16.88	Yes	****	<0.0001
FSam:60 d vs. FSol:40 d	8.5	6.250 to 10.75	Yes	****	<0.0001
FSam:60 d vs. FSol:60 d	-0.1667	-2.417 to 2.084	No	ns	>0.9999
FSam:60 d vs. FSam+FSol:0 d	24.87	22.62 to 27.12	Yes	****	<0.0001
FSam:60 d vs. FSam+FSol:20 d	11.9	9.650 to 14.15	Yes	****	<0.0001
FSam:60 d vs. FSam+FSol:40 d	3.267	1.016 to 5.517	Yes	***	0.0006

FSam:60 d vs. FSam+FSol:60 d	-8.433	-10.68 to -6.183	Yes	****	<0.0001
FSol:0 d vs. FSol:20 d	-10.23	-12.48 to -7.983	Yes	****	<0.0001
FSol:0 d vs. FSol:40 d	-16.37	-18.62 to -14.12	Yes	****	<0.0001
FSol:0 d vs. FSol:60 d	-25.03	-27.28 to -22.78	Yes	****	<0.0001
FSol:0 d vs. FSam+FSol:0 d	5.33E-15	-2.250 to 2.250	No	ns	>0.9999
FSol:0 d vs. FSam+FSol:20 d	-12.97	-15.22 to -10.72	Yes	****	<0.0001
FSol:0 d vs. FSam+FSol:40 d	-21.6	-23.85 to -19.35	Yes	****	<0.0001
FSol:0 d vs. FSam+FSol:60 d	-33.3	-35.55 to -31.05	Yes	****	<0.0001
FSol:20 d vs. FSol:40 d	-6.133	-8.384 to -3.883	Yes	****	<0.0001
FSol:20 d vs. FSol:60 d	-14.8	-17.05 to -12.55	Yes	****	<0.0001
FSol:20 d vs. FSam+FSol:0 d	10.23	7.983 to 12.48	Yes	****	<0.0001
FSol:20 d vs. FSam+FSol:20 d	-2.733	-4.984 to -0.4829	Yes	**	0.0067
FSol:20 d vs. FSam+FSol:40 d	-11.37	-13.62 to -9.116	Yes	****	<0.0001
FSol:20 d vs. FSam+FSol:60 d	-23.07	-25.32 to -20.82	Yes	****	<0.0001
FSol:40 d vs. FSol:60 d	-8.667	-10.92 to -6.416	Yes	****	<0.0001
FSol:40 d vs. FSam+FSol:0 d	16.37	14.12 to 18.62	Yes	****	<0.0001
FSol:40 d vs. FSam+FSol:20 d	3.4	1.150 to 5.650	Yes	***	0.0003
FSol:40 d vs. FSam+FSol:40 d	-5.233	-7.484 to -2.983	Yes	****	<0.0001
FSol:40 d vs. FSam+FSol:60 d	-16.93	-19.18 to -14.68	Yes	****	<0.0001
FSol:60 d vs. FSam+FSol:0 d	25.03	22.78 to 27.28	Yes	****	<0.0001
FSol:60 d vs. FSam+FSol:20 d	12.07	9.816 to 14.32	Yes	****	<0.0001
FSol:60 d vs. FSam+FSol:40 d	3.433	1.183 to 5.684	Yes	***	0.0003
FSol:60 d vs. FSam+FSol:60 d	-8.267	-10.52 to -6.016	Yes	****	<0.0001
FSam+FSol:0 d vs. FSam+FSol:20 d	-12.97	-15.22 to -10.72	Yes	****	<0.0001
FSam+FSol:0 d vs. FSam+FSol:40 d	-21.6	-23.85 to -19.35	Yes	****	<0.0001
FSam+FSol:0 d vs. FSam+FSol:60 d	-33.3	-35.55 to -31.05	Yes	****	<0.0001
FSam+FSol:20 d vs. FSam+FSol:40 d	-8.633	-10.88 to -6.383	Yes	****	<0.0001
FSam+FSol:20 d vs. FSam+FSol:60 d	-20.33	-22.58 to -18.08	Yes	****	<0.0001
FSam+FSol:40 d vs. FSam+FSol:60 d	-11.7	-13.95 to -9.450	Yes	****	<0.0001

Table S3: Tukey multiple comparison test for lesion depth of Kufri Jyoti when infected with control (water agar), *Fusarium Sambucinum*, *Fusarium solani* and *Fusarium Sambucinum* + *Fusarium solani* and incubated for 0 d, 20 d, 40 d and 60 days

Tukey's multiple comparisons test	Mean Diff.	95.00% CI of diff.	Significant?	Summary	Adjusted P Value
Control:0 d vs. Control:20 d	-3.6E-15	-2.400 to 2.400	No	ns	>0.9999
Control:0 d vs. Control:40 d	-7.1E-15	-2.400 to 2.400	No	ns	>0.9999
Control:0 d vs. Control:60 d	-7.1E-15	-2.400 to 2.400	No	ns	>0.9999
Control:0 d vs. FSam:0 d	-7.1E-15	-2.400 to 2.400	No	ns	>0.9999
Control:0 d vs. FSam:20 d	-7.567	-9.967 to -5.166	Yes	****	<0.0001
Control:0 d vs. FSam:40 d	-14.3	-16.70 to -11.90	Yes	****	<0.0001
Control:0 d vs. FSam:60 d	-22.9	-25.30 to -20.50	Yes	****	<0.0001
Control:0 d vs. FSol:0 d	-7.1E-15	-2.400 to 2.400	No	ns	>0.9999
Control:0 d vs. FSol:20 d	-5.233	-7.634 to -2.833	Yes	****	<0.0001
Control:0 d vs. FSol:40 d	-9	-11.40 to -6.600	Yes	****	<0.0001
Control:0 d vs. FSol:60 d	-14.53	-16.93 to -12.13	Yes	****	<0.0001
Control:0 d vs. FSam+FSol:0 d	-7.1E-15	-2.400 to 2.400	No	ns	>0.9999
Control:0 d vs. FSam+FSol:20 d	-11.43	-13.83 to -9.033	Yes	****	<0.0001
Control:0 d vs. FSam+FSol:40 d	-18.03	-20.43 to -15.63	Yes	****	<0.0001
Control:0 d vs. FSam+FSol:60 d	-29.1	-31.50 to -26.70	Yes	****	<0.0001
Control:20 d vs. Control:40 d	-3.6E-15	-2.400 to 2.400	No	ns	>0.9999
Control:20 d vs. Control:60 d	-3.6E-15	-2.400 to 2.400	No	ns	>0.9999
Control:20 d vs. FSam:0 d	-3.6E-15	-2.400 to 2.400	No	ns	>0.9999
Control:20 d vs. FSam:20 d	-7.567	-9.967 to -5.166	Yes	****	<0.0001
Control:20 d vs. FSam:40 d	-14.3	-16.70 to -11.90	Yes	****	<0.0001
Control:20 d vs. FSam:60 d	-22.9	-25.30 to -20.50	Yes	****	<0.0001
Control:20 d vs. FSol:0 d	-3.6E-15	-2.400 to 2.400	No	ns	>0.9999

Control:20 d vs. FSol:20 d	-5.233	-7.634 to -2.833	Yes	****	<0.0001
Control:20 d vs. FSol:40 d	-9	-11.40 to -6.600	Yes	****	<0.0001
Control:20 d vs. FSol:60 d	-14.53	-16.93 to -12.13	Yes	****	<0.0001
Control:20 d vs. FSam+FSol:0 d	-3.6E-15	-2.400 to 2.400	No	ns	>0.9999
Control:20 d vs. FSam+FSol:20 d	-11.43	-13.83 to -9.033	Yes	****	<0.0001
Control:20 d vs. FSam+FSol:40 d	-18.03	-20.43 to -15.63	Yes	****	<0.0001
Control:20 d vs. FSam+FSol:60 d	-29.1	-31.50 to -26.70	Yes	****	<0.0001
Control:40 d vs. Control:60 d	0	-2.400 to 2.400	No	ns	>0.9999
Control:40 d vs. FSam:0 d	0	-2.400 to 2.400	No	ns	>0.9999
Control:40 d vs. FSam:20 d	-7.567	-9.967 to -5.166	Yes	****	<0.0001
Control:40 d vs. FSam:40 d	-14.3	-16.70 to -11.90	Yes	****	<0.0001
Control:40 d vs. FSam:60 d	-22.9	-25.30 to -20.50	Yes	****	<0.0001
Control:40 d vs. FSol:0 d	0	-2.400 to 2.400	No	ns	>0.9999
Control:40 d vs. FSol:20 d	-5.233	-7.634 to -2.833	Yes	****	<0.0001
Control:40 d vs. FSol:40 d	-9	-11.40 to -6.600	Yes	****	<0.0001
Control:40 d vs. FSol:60 d	-14.53	-16.93 to -12.13	Yes	****	<0.0001
Control:40 d vs. FSam+FSol:0 d	0	-2.400 to 2.400	No	ns	>0.9999
Control:40 d vs. FSam+FSol:20 d	-11.43	-13.83 to -9.033	Yes	****	<0.0001
Control:40 d vs. FSam+FSol:40 d	-18.03	-20.43 to -15.63	Yes	****	<0.0001
Control:40 d vs. FSam+FSol:60 d	-29.1	-31.50 to -26.70	Yes	****	<0.0001
Control:60 d vs. FSam:0 d	0	-2.400 to 2.400	No	ns	>0.9999
Control:60 d vs. FSam:20 d	-7.567	-9.967 to -5.166	Yes	****	<0.0001
Control:60 d vs. FSam:40 d	-14.3	-16.70 to -11.90	Yes	****	<0.0001
Control:60 d vs. FSam:60 d	-22.9	-25.30 to -20.50	Yes	****	<0.0001
Control:60 d vs. FSol:0 d	0	-2.400 to 2.400	No	ns	>0.9999
Control:60 d vs. FSol:20 d	-5.233	-7.634 to -2.833	Yes	****	<0.0001
Control:60 d vs. FSol:40 d	-9	-11.40 to -6.600	Yes	****	<0.0001
Control:60 d vs. FSol:60 d	-14.53	-16.93 to -12.13	Yes	****	<0.0001
Control:60 d vs. FSam+FSol:0 d	0	-2.400 to 2.400	No	ns	>0.9999

Control:60 d vs. FSam+FSol:20 d	-11.43	-13.83 to -9.033	Yes	****	<0.0001
Control:60 d vs. FSam+FSol:40 d	-18.03	-20.43 to -15.63	Yes	****	<0.0001
Control:60 d vs. FSam+FSol:60 d	-29.1	-31.50 to -26.70	Yes	****	<0.0001
FSam:0 d vs. FSam:20 d	-7.567	-9.967 to -5.166	Yes	****	<0.0001
FSam:0 d vs. FSam:40 d	-14.3	-16.70 to -11.90	Yes	****	<0.0001
FSam:0 d vs. FSam:60 d	-22.9	-25.30 to -20.50	Yes	****	<0.0001
FSam:0 d vs. FSol:0 d	0	-2.400 to 2.400	No	ns	>0.9999
FSam:0 d vs. FSol:20 d	-5.233	-7.634 to -2.833	Yes	****	<0.0001
FSam:0 d vs. FSol:40 d	-9	-11.40 to -6.600	Yes	****	<0.0001
FSam:0 d vs. FSol:60 d	-14.53	-16.93 to -12.13	Yes	****	<0.0001
FSam:0 d vs. FSam+FSol:0 d	0	-2.400 to 2.400	No	ns	>0.9999
FSam:0 d vs. FSam+FSol:20 d	-11.43	-13.83 to -9.033	Yes	****	<0.0001
FSam:0 d vs. FSam+FSol:40 d	-18.03	-20.43 to -15.63	Yes	****	<0.0001
FSam:0 d vs. FSam+FSol:60 d	-29.1	-31.50 to -26.70	Yes	****	<0.0001
FSam:20 d vs. FSam:40 d	-6.733	-9.134 to -4.333	Yes	****	<0.0001
FSam:20 d vs. FSam:60 d	-15.33	-17.73 to -12.93	Yes	****	<0.0001
FSam:20 d vs. FSol:0 d	7.567	5.166 to 9.967	Yes	****	<0.0001
FSam:20 d vs. FSol:20 d	2.333	-0.06700 to 4.734	No	ns	0.0636
FSam:20 d vs. FSol:40 d	-1.433	-3.834 to 0.9670	No	ns	0.6818
FSam:20 d vs. FSol:60 d	-6.967	-9.367 to -4.566	Yes	****	<0.0001
FSam:20 d vs. FSam+FSol:0 d	7.567	5.166 to 9.967	Yes	****	<0.0001
FSam:20 d vs. FSam+FSol:20 d	-3.867	-6.267 to -1.466	Yes	***	0.0001
FSam:20 d vs. FSam+FSol:40 d	-10.47	-12.87 to -8.066	Yes	****	<0.0001
FSam:20 d vs. FSam+FSol:60 d	-21.53	-23.93 to -19.13	Yes	****	<0.0001
FSam:40 d vs. FSam:60 d	-8.6	-11.00 to -6.200	Yes	****	<0.0001
FSam:40 d vs. FSol:0 d	14.3	11.90 to 16.70	Yes	****	<0.0001
FSam:40 d vs. FSol:20 d	9.067	6.666 to 11.47	Yes	****	<0.0001
FSam:40 d vs. FSol:40 d	5.3	2.900 to 7.700	Yes	****	<0.0001
FSam:40 d vs. FSol:60 d	-0.2333	-2.634 to 2.167	No	ns	>0.9999
FSam:40 d vs. FSam+FSol:0 d	14.3	11.90 to 16.70	Yes	****	<0.0001
FSam:40 d vs. FSam+FSol:20 d	2.867	0.4663 to 5.267	Yes	**	0.0082

FSam:40 d vs. FSam+FSol:40 d	-3.733	-6.134 to -1.333	Yes	***	0.0002
FSam:40 d vs. FSam+FSol:60 d	-14.8	-17.20 to -12.40	Yes	****	<0.0001
FSam:60 d vs. FSol:0 d	22.9	20.50 to 25.30	Yes	****	<0.0001
FSam:60 d vs. FSol:20 d	17.67	15.27 to 20.07	Yes	****	<0.0001
FSam:60 d vs. FSol:40 d	13.9	11.50 to 16.30	Yes	****	<0.0001
FSam:60 d vs. FSol:60 d	8.367	5.966 to 10.77	Yes	****	<0.0001
FSam:60 d vs. FSam+FSol:0 d	22.9	20.50 to 25.30	Yes	****	<0.0001
FSam:60 d vs. FSam+FSol:20 d	11.47	9.066 to 13.87	Yes	****	<0.0001
FSam:60 d vs. FSam+FSol:40 d	4.867	2.466 to 7.267	Yes	****	<0.0001
FSam:60 d vs. FSam+FSol:60 d	-6.2	-8.600 to -3.800	Yes	****	<0.0001
FSol:0 d vs. FSol:20 d	-5.233	-7.634 to -2.833	Yes	****	<0.0001
FSol:0 d vs. FSol:40 d	-9	-11.40 to -6.600	Yes	****	<0.0001
FSol:0 d vs. FSol:60 d	-14.53	-16.93 to -12.13	Yes	****	<0.0001
FSol:0 d vs. FSam+FSol:0 d	0	-2.400 to 2.400	No	ns	>0.9999
FSol:0 d vs. FSam+FSol:20 d	-11.43	-13.83 to -9.033	Yes	****	<0.0001
FSol:0 d vs. FSam+FSol:40 d	-18.03	-20.43 to -15.63	Yes	****	<0.0001
FSol:0 d vs. FSam+FSol:60 d	-29.1	-31.50 to -26.70	Yes	****	<0.0001
FSol:20 d vs. FSol:40 d	-3.767	-6.167 to -1.366	Yes	***	0.0002
FSol:20 d vs. FSol:60 d	-9.3	-11.70 to -6.900	Yes	****	<0.0001
FSol:20 d vs. FSam+FSol:0 d	5.233	2.833 to 7.634	Yes	****	<0.0001
FSol:20 d vs. FSam+FSol:20 d	-6.2	-8.600 to -3.800	Yes	****	<0.0001
FSol:20 d vs. FSam+FSol:40 d	-12.8	-15.20 to -10.40	Yes	****	<0.0001
FSol:20 d vs. FSam+FSol:60 d	-23.87	-26.27 to -21.47	Yes	****	<0.0001
FSol:40 d vs. FSol:60 d	-5.533	-7.934 to -3.133	Yes	****	<0.0001
FSol:40 d vs. FSam+FSol:0 d	9	6.600 to 11.40	Yes	****	<0.0001
FSol:40 d vs. FSam+FSol:20 d	-2.433	-4.834 to -0.03300	Yes	*	0.0443
FSol:40 d vs. FSam+FSol:40 d	-9.033	-11.43 to -6.633	Yes	****	<0.0001
FSol:40 d vs. FSam+FSol:60 d	-20.1	-22.50 to -17.70	Yes	****	<0.0001
FSol:60 d vs. FSam+FSol:0 d	14.53	12.13 to 16.93	Yes	****	<0.0001
FSol:60 d vs. FSam+FSol:20 d	3.1	0.6997 to 5.500	Yes	**	0.0031
FSol:60 d vs. FSam+FSol:40 d	-3.5	-5.900 to -1.100	Yes	***	0.0006
FSol:60 d vs. FSam+FSol:60 d	-14.57	-16.97 to -12.17	Yes	****	<0.0001

FSam+FSol:0 d vs. FSam+FSol:20 d	-11.43	-13.83 to -9.033	Yes	****	<0.0001
FSam+FSol:0 d vs. FSam+FSol:40 d	-18.03	-20.43 to -15.63	Yes	****	<0.0001
FSam+FSol:0 d vs. FSam+FSol:60 d	-29.1	-31.50 to -26.70	Yes	****	<0.0001
FSam+FSol:20 d vs. FSam+FSol:40 d	-6.6	-9.000 to -4.200	Yes	****	<0.0001
FSam+FSol:20 d vs. FSam+FSol:60 d	-17.67	-20.07 to -15.27	Yes	****	<0.0001
FSam+FSol:40 d vs. FSam+FSol:60 d	-11.07	-13.47 to -8.666	Yes	****	<0.0001

Table S4: Tukey multiple comparison test for lesion depth of Kufri Frysona when infected with control (water agar), *Fusarium Sambucinum*, *Fusarium solani* and *Fusarium Sambucinum* + *Fusarium solani* and incubated for 0 d, 20 d, 40 d and 60 days

Tukey's multiple comparisons test	Mean Diff.	95.00% CI of diff.	Significant?	Summary	Adjusted P Value
Control:0 d vs. Control:20 d	0	-2.016 to 2.016	No	ns	>0.9999
Control:0 d vs. Control:40 d	0	-2.016 to 2.016	No	ns	>0.9999
Control:0 d vs. Control:60 d	-7.1E-15	-2.016 to 2.016	No	ns	>0.9999
Control:0 d vs. FSam:0 d	3.55E-15	-2.016 to 2.016	No	ns	>0.9999
Control:0 d vs. FSam:20 d	-6.1	-8.116 to -4.084	Yes	****	<0.0001
Control:0 d vs. FSam:40 d	-11.8	-13.82 to -9.784	Yes	****	<0.0001
Control:0 d vs. FSam:60 d	-18.03	-20.05 to -16.02	Yes	****	<0.0001
Control:0 d vs. FSol:0 d	0	-2.016 to 2.016	No	ns	>0.9999
Control:0 d vs. FSol:20 d	-4.367	-6.383 to -2.350	Yes	****	<0.0001
Control:0 d vs. FSol:40 d	-7.1	-9.116 to -5.084	Yes	****	<0.0001
Control:0 d vs. FSol:60 d	-9.4	-11.42 to -7.384	Yes	****	<0.0001
Control:0 d vs. FSam+FSol:0 d	0	-2.016 to 2.016	No	ns	>0.9999
Control:0 d vs. FSam+FSol:20 d	-8.033	-10.05 to -6.017	Yes	****	<0.0001
Control:0 d vs. FSam+FSol:40 d	-15.33	-17.35 to -13.32	Yes	****	<0.0001
Control:0 d vs. FSam+FSol:60 d	-21.73	-23.75 to -19.72	Yes	****	<0.0001
Control:20 d vs. Control:40 d	0	-2.016 to 2.016	No	ns	>0.9999

Control:20 d vs. Control:60 d	-7.1E-15	-2.016 to 2.016	No	ns	>0.9999
Control:20 d vs. FSam:0 d	3.55E-15	-2.016 to 2.016	No	ns	>0.9999
Control:20 d vs. FSam:20 d	-6.1	-8.116 to -4.084	Yes	****	<0.0001
Control:20 d vs. FSam:40 d	-11.8	-13.82 to -9.784	Yes	****	<0.0001
Control:20 d vs. FSam:60 d	-18.03	-20.05 to -16.02	Yes	****	<0.0001
Control:20 d vs. FSol:0 d	0	-2.016 to 2.016	No	ns	>0.9999
Control:20 d vs. FSol:20 d	-4.367	-6.383 to -2.350	Yes	****	<0.0001
Control:20 d vs. FSol:40 d	-7.1	-9.116 to -5.084	Yes	****	<0.0001
Control:20 d vs. FSol:60 d	-9.4	-11.42 to -7.384	Yes	****	<0.0001
Control:20 d vs. FSam+FSol:0 d	0	-2.016 to 2.016	No	ns	>0.9999
Control:20 d vs. FSam+FSol:20 d	-8.033	-10.05 to -6.017	Yes	****	<0.0001
Control:20 d vs. FSam+FSol:40 d	-15.33	-17.35 to -13.32	Yes	****	<0.0001
Control:20 d vs. FSam+FSol:60 d	-21.73	-23.75 to -19.72	Yes	****	<0.0001
Control:40 d vs. Control:60 d	-7.1E-15	-2.016 to 2.016	No	ns	>0.9999
Control:40 d vs. FSam:0 d	3.55E-15	-2.016 to 2.016	No	ns	>0.9999
Control:40 d vs. FSam:20 d	-6.1	-8.116 to -4.084	Yes	****	<0.0001
Control:40 d vs. FSam:40 d	-11.8	-13.82 to -9.784	Yes	****	<0.0001
Control:40 d vs. FSam:60 d	-18.03	-20.05 to -16.02	Yes	****	<0.0001
Control:40 d vs. FSol:0 d	0	-2.016 to 2.016	No	ns	>0.9999
Control:40 d vs. FSol:20 d	-4.367	-6.383 to -2.350	Yes	****	<0.0001
Control:40 d vs. FSol:40 d	-7.1	-9.116 to -5.084	Yes	****	<0.0001
Control:40 d vs. FSol:60 d	-9.4	-11.42 to -7.384	Yes	****	<0.0001
Control:40 d vs. FSam+FSol:0 d	0	-2.016 to 2.016	No	ns	>0.9999
Control:40 d vs. FSam+FSol:20 d	-8.033	-10.05 to -6.017	Yes	****	<0.0001
Control:40 d vs. FSam+FSol:40 d	-15.33	-17.35 to -13.32	Yes	****	<0.0001
Control:40 d vs. FSam+FSol:60 d	-21.73	-23.75 to -19.72	Yes	****	<0.0001
Control:60 d vs. FSam:0 d	1.07E-14	-2.016 to 2.016	No	ns	>0.9999

Control:60 d vs. FSam:20 d	-6.1	-8.116 to -4.084	Yes	****	<0.0001
Control:60 d vs. FSam:40 d	-11.8	-13.82 to -9.784	Yes	****	<0.0001
Control:60 d vs. FSam:60 d	-18.03	-20.05 to -16.02	Yes	****	<0.0001
Control:60 d vs. FSol:0 d	7.11E-15	-2.016 to 2.016	No	ns	>0.9999
Control:60 d vs. FSol:20 d	-4.367	-6.383 to -2.350	Yes	****	<0.0001
Control:60 d vs. FSol:40 d	-7.1	-9.116 to -5.084	Yes	****	<0.0001
Control:60 d vs. FSol:60 d	-9.4	-11.42 to -7.384	Yes	****	<0.0001
Control:60 d vs. FSam+FSol:0 d	7.11E-15	-2.016 to 2.016	No	ns	>0.9999
Control:60 d vs. FSam+FSol:20 d	-8.033	-10.05 to -6.017	Yes	****	<0.0001
Control:60 d vs. FSam+FSol:40 d	-15.33	-17.35 to -13.32	Yes	****	<0.0001
Control:60 d vs. FSam+FSol:60 d	-21.73	-23.75 to -19.72	Yes	****	<0.0001
FSam:0 d vs. FSam:20 d	-6.1	-8.116 to -4.084	Yes	****	<0.0001
FSam:0 d vs. FSam:40 d	-11.8	-13.82 to -9.784	Yes	****	<0.0001
FSam:0 d vs. FSam:60 d	-18.03	-20.05 to -16.02	Yes	****	<0.0001
FSam:0 d vs. FSol:0 d	-3.6E-15	-2.016 to 2.016	No	ns	>0.9999
FSam:0 d vs. FSol:20 d	-4.367	-6.383 to -2.350	Yes	****	<0.0001
FSam:0 d vs. FSol:40 d	-7.1	-9.116 to -5.084	Yes	****	<0.0001
FSam:0 d vs. FSol:60 d	-9.4	-11.42 to -7.384	Yes	****	<0.0001
FSam:0 d vs. FSam+FSol:0 d	-3.6E-15	-2.016 to 2.016	No	ns	>0.9999
FSam:0 d vs. FSam+FSol:20 d	-8.033	-10.05 to -6.017	Yes	****	<0.0001
FSam:0 d vs. FSam+FSol:40 d	-15.33	-17.35 to -13.32	Yes	****	<0.0001
FSam:0 d vs. FSam+FSol:60 d	-21.73	-23.75 to -19.72	Yes	****	<0.0001
FSam:20 d vs. FSam:40 d	-5.7	-7.716 to -3.684	Yes	****	<0.0001
FSam:20 d vs. FSam:60 d	-11.93	-13.95 to -9.917	Yes	****	<0.0001
FSam:20 d vs. FSol:0 d	6.1	4.084 to 8.116	Yes	****	<0.0001
FSam:20 d vs. FSol:20 d	1.733	-0.2830 to 3.750	No	ns	0.1563
FSam:20 d vs. FSol:40 d	-1	-3.016 to 1.016	No	ns	0.8837

FSam:20 d vs. FSol:60 d	-3.3	-5.316 to -1.284	Yes	****	<0.0001
FSam:20 d vs. FSam+FSol:0 d	6.1	4.084 to 8.116	Yes	****	<0.0001
FSam:20 d vs. FSam+FSol:20 d	-1.933	-3.950 to 0.08304	No	ns	0.0711
FSam:20 d vs. FSam+FSol:40 d	-9.233	-11.25 to -7.217	Yes	****	<0.0001
FSam:20 d vs. FSam+FSol:60 d	-15.63	-17.65 to -13.62	Yes	****	<0.0001
FSam:40 d vs. FSam:60 d	-6.233	-8.250 to -4.217	Yes	****	<0.0001
FSam:40 d vs. FSol:0 d	11.8	9.784 to 13.82	Yes	****	<0.0001
FSam:40 d vs. FSol:20 d	7.433	5.417 to 9.450	Yes	****	<0.0001
FSam:40 d vs. FSol:40 d	4.7	2.684 to 6.716	Yes	****	<0.0001
FSam:40 d vs. FSol:60 d	2.4	0.3836 to 4.416	Yes	**	0.0085
FSam:40 d vs. FSam+FSol:0 d	11.8	9.784 to 13.82	Yes	****	<0.0001
FSam:40 d vs. FSam+FSol:20 d	3.767	1.750 to 5.783	Yes	****	<0.0001
FSam:40 d vs. FSam+FSol:40 d	-3.533	-5.550 to -1.517	Yes	****	<0.0001
FSam:40 d vs. FSam+FSol:60 d	-9.933	-11.95 to -7.917	Yes	****	<0.0001
FSam:60 d vs. FSol:0 d	18.03	16.02 to 20.05	Yes	****	<0.0001
FSam:60 d vs. FSol:20 d	13.67	11.65 to 15.68	Yes	****	<0.0001
FSam:60 d vs. FSol:40 d	10.93	8.917 to 12.95	Yes	****	<0.0001
FSam:60 d vs. FSol:60 d	8.633	6.617 to 10.65	Yes	****	<0.0001
FSam:60 d vs. FSam+FSol:0 d	18.03	16.02 to 20.05	Yes	****	<0.0001
FSam:60 d vs. FSam+FSol:20 d	10	7.984 to 12.02	Yes	****	<0.0001
FSam:60 d vs. FSam+FSol:40 d	2.7	0.6836 to 4.716	Yes	**	0.0019
FSam:60 d vs. FSam+FSol:60 d	-3.7	-5.716 to -1.684	Yes	****	<0.0001
FSol:0 d vs. FSol:20 d	-4.367	-6.383 to -2.350	Yes	****	<0.0001
FSol:0 d vs. FSol:40 d	-7.1	-9.116 to -5.084	Yes	****	<0.0001
FSol:0 d vs. FSol:60 d	-9.4	-11.42 to -7.384	Yes	****	<0.0001
FSol:0 d vs. FSam+FSol:0 d	0	-2.016 to 2.016	No	ns	>0.9999
FSol:0 d vs. FSam+FSol:20 d	-8.033	-10.05 to -6.017	Yes	****	<0.0001
FSol:0 d vs. FSam+FSol:40 d	-15.33	-17.35 to -13.32	Yes	****	<0.0001
FSol:0 d vs. FSam+FSol:60 d	-21.73	-23.75 to -19.72	Yes	****	<0.0001
FSol:20 d vs. FSol:40 d	-2.733	-4.750 to -0.7170	Yes	**	0.0016
FSol:20 d vs. FSol:60 d	-5.033	-7.050 to -3.017	Yes	****	<0.0001
FSol:20 d vs. FSam+FSol:0 d	4.367	2.350 to 6.383	Yes	****	<0.0001

FSol:20 d vs. FSam+FSol:20 d	-3.667	-5.683 to -1.650	Yes	****	<0.0001
FSol:20 d vs. FSam+FSol:40 d	-10.97	-12.98 to -8.950	Yes	****	<0.0001
FSol:20 d vs. FSam+FSol:60 d	-17.37	-19.38 to -15.35	Yes	****	<0.0001
FSol:40 d vs. FSol:60 d	-2.3	-4.316 to -0.2836	Yes	*	0.0138
FSol:40 d vs. FSam+FSol:0 d	7.1	5.084 to 9.116	Yes	****	<0.0001
FSol:40 d vs. FSam+FSol:20 d	-0.9333	-2.950 to 1.083	No	ns	0.927
FSol:40 d vs. FSam+FSol:40 d	-8.233	-10.25 to -6.217	Yes	****	<0.0001
FSol:40 d vs. FSam+FSol:60 d	-14.63	-16.65 to -12.62	Yes	****	<0.0001
FSol:60 d vs. FSam+FSol:0 d	9.4	7.384 to 11.42	Yes	****	<0.0001
FSol:60 d vs. FSam+FSol:20 d	1.367	-0.6497 to 3.383	No	ns	0.4867
FSol:60 d vs. FSam+FSol:40 d	-5.933	-7.950 to -3.917	Yes	****	<0.0001
FSol:60 d vs. FSam+FSol:60 d	-12.33	-14.35 to -10.32	Yes	****	<0.0001
FSam+FSol:0 d vs. FSam+FSol:20 d	-8.033	-10.05 to -6.017	Yes	****	<0.0001
FSam+FSol:0 d vs. FSam+FSol:40 d	-15.33	-17.35 to -13.32	Yes	****	<0.0001
FSam+FSol:0 d vs. FSam+FSol:60 d	-21.73	-23.75 to -19.72	Yes	****	<0.0001
FSam+FSol:20 d vs. FSam+FSol:40 d	-7.3	-9.316 to -5.284	Yes	****	<0.0001
FSam+FSol:20 d vs. FSam+FSol:60 d	-13.7	-15.72 to -11.68	Yes	****	<0.0001
FSam+FSol:40 d vs. FSam+FSol:60 d	-6.4	-8.416 to -4.384	Yes	****	<0.0001

Table S5: Bonferroni's multiple comparisons test for rot volume of Kufri Jyoti and Kufri Frysona when infected with control (water agar), *Fusarium Sambucinum*, *Fusarium solani* and *Fusarium Sambucinum* + *Fusarium solani* and incubated for 0 d, 20 d, 40 d and 60 days

Bonferroni's multiple comparisons test	Mean Diff.	95.00% CI of diff.	Significant?	Summary	Adjusted P Value
Control:0 d vs. Control:20 d	0	-2592 to 2592	No	ns	>0.9999
Control:0 d vs. Control:40 d	-1.819E-12	-2592 to 2592	No	ns	>0.9999
Control:0 d vs. Control:60 d	0	-2592 to 2592	No	ns	>0.9999
Control:0 d vs. FSam:0 d	5.457E-12	-2592 to 2592	No	ns	>0.9999
Control:0 d vs. FSam:20 d	-1120	-3712 to 1472	No	ns	>0.9999
Control:0 d vs. FSam:40 d	-7259	-9851 to -4667	Yes	****	<0.0001

Control:0 d vs. FSam:60 d	-28200	-30792 to -25608	Yes	****	<0.0001
Control:0 d vs. FSol:0 d	0	-2592 to 2592	No	ns	>0.9999
Control:0 d vs. FSol:20 d	-581.9	-3174 to 2010	No	ns	>0.9999
Control:0 d vs. FSol:40 d	-3055	-5647 to -463.2	Yes	**	0.0035
Control:0 d vs. FSol:60 d	-8667	-11259 to -6075	Yes	****	<0.0001
Control:0 d vs. FSam+FSol:0 d	0	-2592 to 2592	No	ns	>0.9999
Control:0 d vs. FSam+FSol:20 d	-2050	-4642 to 542.4	No	ns	0.8344
Control:0 d vs. FSam+FSol:40 d	-10941	-13534 to -8349	Yes	****	<0.0001
Control:0 d vs. FSam+FSol:60 d	-38056	-40648 to -35464	Yes	****	<0.0001
Control:0 d vs. Control:0 d	0	-2592 to 2592	No	ns	>0.9999
Control:0 d vs. Control:20 d	3.638E-12	-2592 to 2592	No	ns	>0.9999
Control:0 d vs. Control:40 d	-1.819E-12	-2592 to 2592	No	ns	>0.9999
Control:0 d vs. Control:60 d	0	-2592 to 2592	No	ns	>0.9999
Control:0 d vs. FSam:0 d	2.728E-12	-2592 to 2592	No	ns	>0.9999
Control:0 d vs. FSam:20 d	-719.2	-3311 to 1873	No	ns	>0.9999
Control:0 d vs. FSam:40 d	-3745	-6337 to -1153	Yes	****	<0.0001
Control:0 d vs. FSam:60 d	-8763	-11355 to -6171	Yes	****	<0.0001
Control:0 d vs. FSol:0 d	-2.728E-12	-2592 to 2592	No	ns	>0.9999
Control:0 d vs. FSol:20 d	-359.3	-2951 to 2233	No	ns	>0.9999
Control:0 d vs. FSol:40 d	-1488	-4080 to 1104	No	ns	>0.9999
Control:0 d vs. FSol:60 d	-4608	-7200 to -2016	Yes	****	<0.0001
Control:0 d vs. FSam+FSol:0 d	0	-2592 to 2592	No	ns	>0.9999
Control:0 d vs. FSam+FSol:20 d	-1061	-3653 to 1531	No	ns	>0.9999
Control:0 d vs. FSam+FSol:40 d	-5606	-8198 to -3014	Yes	****	<0.0001
Control:0 d vs. FSam+FSol:60 d	-19013	-21605 to -16421	Yes	****	<0.0001
Control:20 d vs. Control:40 d	-1.819E-12	-2592 to 2592	No	ns	>0.9999
Control:20 d vs. Control:60 d	0	-2592 to 2592	No	ns	>0.9999
Control:20 d vs. FSam:0 d	5.457E-12	-2592 to 2592	No	ns	>0.9999
Control:20 d vs. FSam:20 d	-1120	-3712 to 1472	No	ns	>0.9999
Control:20 d vs. FSam:40 d	-7259	-9851 to -4667	Yes	****	<0.0001

Control:20 d vs. FSam:60 d	-28200	-30792 to -25608	Yes	****	<0.0001
Control:20 d vs. FSol:0 d	0	-2592 to 2592	No	ns	>0.9999
Control:20 d vs. FSol:20 d	-581.9	-3174 to 2010	No	ns	>0.9999
Control:20 d vs. FSol:40 d	-3055	-5647 to -463.2	Yes	**	0.0035
Control:20 d vs. FSol:60 d	-8667	-11259 to -6075	Yes	****	<0.0001
Control:20 d vs. FSam+FSol:0 d	0	-2592 to 2592	No	ns	>0.9999
Control:20 d vs. FSam+FSol:20 d	-2050	-4642 to 542.4	No	ns	0.8344
Control:20 d vs. FSam+FSol:40 d	-10941	-13534 to -8349	Yes	****	<0.0001
Control:20 d vs. FSam+FSol:60 d	-38056	-40648 to -35464	Yes	****	<0.0001
Control:20 d vs. Control:0 d	0	-2592 to 2592	No	ns	>0.9999
Control:20 d vs. Control:20 d	3.638E-12	-2592 to 2592	No	ns	>0.9999
Control:20 d vs. Control:40 d	-1.819E-12	-2592 to 2592	No	ns	>0.9999
Control:20 d vs. Control:60 d	0	-2592 to 2592	No	ns	>0.9999
Control:20 d vs. FSam:0 d	2.728E-12	-2592 to 2592	No	ns	>0.9999
Control:20 d vs. FSam:20 d	-719.2	-3311 to 1873	No	ns	>0.9999
Control:20 d vs. FSam:40 d	-3745	-6337 to -1153	Yes	****	<0.0001
Control:20 d vs. FSam:60 d	-8763	-11355 to -6171	Yes	****	<0.0001
Control:20 d vs. FSol:0 d	-2.728E-12	-2592 to 2592	No	ns	>0.9999
Control:20 d vs. FSol:20 d	-359.3	-2951 to 2233	No	ns	>0.9999
Control:20 d vs. FSol:40 d	-1488	-4080 to 1104	No	ns	>0.9999
Control:20 d vs. FSol:60 d	-4608	-7200 to -2016	Yes	****	<0.0001
Control:20 d vs. FSam+FSol:0 d	0	-2592 to 2592	No	ns	>0.9999
Control:20 d vs. FSam+FSol:20 d	-1061	-3653 to 1531	No	ns	>0.9999
Control:20 d vs. FSam+FSol:40 d	-5606	-8198 to -3014	Yes	****	<0.0001
Control:20 d vs. FSam+FSol:60 d	-19013	-21605 to -16421	Yes	****	<0.0001
Control:40 d vs. Control:60 d	1.819E-12	-2592 to 2592	No	ns	>0.9999
Control:40 d vs. FSam:0 d	7.276E-12	-2592 to 2592	No	ns	>0.9999
Control:40 d vs. FSam:20 d	-1120	-3712 to 1472	No	ns	>0.9999
Control:40 d vs. FSam:40 d	-7259	-9851 to -4667	Yes	****	<0.0001
Control:40 d vs. FSam:60 d	-28200	-30792 to -25608	Yes	****	<0.0001

Control:40 d vs. FSol:0 d	1.819E-12	-2592 to 2592	No	ns	>0.9999
Control:40 d vs. FSol:20 d	-581.9	-3174 to 2010	No	ns	>0.9999
Control:40 d vs. FSol:40 d	-3055	-5647 to -463.2	Yes	**	0.0035
Control:40 d vs. FSol:60 d	-8667	-11259 to -6075	Yes	****	<0.0001
Control:40 d vs. FSam+FSol:0 d	1.819E-12	-2592 to 2592	No	ns	>0.9999
Control:40 d vs. FSam+FSol:20 d	-2050	-4642 to 542.4	No	ns	0.8344
Control:40 d vs. FSam+FSol:40 d	-10941	-13534 to -8349	Yes	****	<0.0001
Control:40 d vs. FSam+FSol:60 d	-38056	-40648 to -35464	Yes	****	<0.0001
Control:40 d vs. Control:0 d	1.819E-12	-2592 to 2592	No	ns	>0.9999
Control:40 d vs. Control:20 d	5.457E-12	-2592 to 2592	No	ns	>0.9999
Control:40 d vs. Control:40 d	0	-2592 to 2592	No	ns	>0.9999
Control:40 d vs. Control:60 d	1.819E-12	-2592 to 2592	No	ns	>0.9999
Control:40 d vs. FSam:0 d	4.547E-12	-2592 to 2592	No	ns	>0.9999
Control:40 d vs. FSam:20 d	-719.2	-3311 to 1873	No	ns	>0.9999
Control:40 d vs. FSam:40 d	-3745	-6337 to -1153	Yes	****	<0.0001
Control:40 d vs. FSam:60 d	-8763	-11355 to -6171	Yes	****	<0.0001
Control:40 d vs. FSol:0 d	-9.095E-13	-2592 to 2592	No	ns	>0.9999
Control:40 d vs. FSol:20 d	-359.3	-2951 to 2233	No	ns	>0.9999
Control:40 d vs. FSol:40 d	-1488	-4080 to 1104	No	ns	>0.9999
Control:40 d vs. FSol:60 d	-4608	-7200 to -2016	Yes	****	<0.0001
Control:40 d vs. FSam+FSol:0 d	1.819E-12	-2592 to 2592	No	ns	>0.9999
Control:40 d vs. FSam+FSol:20 d	-1061	-3653 to 1531	No	ns	>0.9999
Control:40 d vs. FSam+FSol:40 d	-5606	-8198 to -3014	Yes	****	<0.0001
Control:40 d vs. FSam+FSol:60 d	-19013	-21605 to -16421	Yes	****	<0.0001
Control:60 d vs. FSam:0 d	5.457E-12	-2592 to 2592	No	ns	>0.9999
Control:60 d vs. FSam:20 d	-1120	-3712 to 1472	No	ns	>0.9999
Control:60 d vs. FSam:40 d	-7259	-9851 to -4667	Yes	****	<0.0001
Control:60 d vs. FSam:60 d	-28200	-30792 to -25608	Yes	****	<0.0001
Control:60 d vs. FSol:0 d	0	-2592 to 2592	No	ns	>0.9999
Control:60 d vs. FSol:20 d	-581.9	-3174 to 2010	No	ns	>0.9999

Control:60 d vs. FSol:40 d	-3055	-5647 to -463.2	Yes	**	0.0035
Control:60 d vs. FSol:60 d	-8667	-11259 to -6075	Yes	****	<0.0001
Control:60 d vs. FSam+FSol:0 d	0	-2592 to 2592	No	ns	>0.9999
Control:60 d vs. FSam+FSol:20 d	-2050	-4642 to 542.4	No	ns	0.8344
Control:60 d vs. FSam+FSol:40 d	-10941	-13534 to -8349	Yes	****	<0.0001
Control:60 d vs. FSam+FSol:60 d	-38056	-40648 to -35464	Yes	****	<0.0001
Control:60 d vs. Control:0 d	0	-2592 to 2592	No	ns	>0.9999
Control:60 d vs. Control:20 d	3.638E-12	-2592 to 2592	No	ns	>0.9999
Control:60 d vs. Control:40 d	-1.819E-12	-2592 to 2592	No	ns	>0.9999
Control:60 d vs. Control:60 d	0	-2592 to 2592	No	ns	>0.9999
Control:60 d vs. FSam:0 d	2.728E-12	-2592 to 2592	No	ns	>0.9999
Control:60 d vs. FSam:20 d	-719.2	-3311 to 1873	No	ns	>0.9999
Control:60 d vs. FSam:40 d	-3745	-6337 to -1153	Yes	****	<0.0001
Control:60 d vs. FSam:60 d	-8763	-11355 to -6171	Yes	****	<0.0001
Control:60 d vs. FSol:0 d	-2.728E-12	-2592 to 2592	No	ns	>0.9999
Control:60 d vs. FSol:20 d	-359.3	-2951 to 2233	No	ns	>0.9999
Control:60 d vs. FSol:40 d	-1488	-4080 to 1104	No	ns	>0.9999
Control:60 d vs. FSol:60 d	-4608	-7200 to -2016	Yes	****	<0.0001
Control:60 d vs. FSam+FSol:0 d	0	-2592 to 2592	No	ns	>0.9999
Control:60 d vs. FSam+FSol:20 d	-1061	-3653 to 1531	No	ns	>0.9999
Control:60 d vs. FSam+FSol:40 d	-5606	-8198 to -3014	Yes	****	<0.0001
Control:60 d vs. FSam+FSol:60 d	-19013	-21605 to -16421	Yes	****	<0.0001
FSam:0 d vs. FSam:20 d	-1120	-3712 to 1472	No	ns	>0.9999
FSam:0 d vs. FSam:40 d	-7259	-9851 to -4667	Yes	****	<0.0001
FSam:0 d vs. FSam:60 d	-28200	-30792 to -25608	Yes	****	<0.0001
FSam:0 d vs. FSol:0 d	-5.457E-12	-2592 to 2592	No	ns	>0.9999
FSam:0 d vs. FSol:20 d	-581.9	-3174 to 2010	No	ns	>0.9999
FSam:0 d vs. FSol:40 d	-3055	-5647 to -463.2	Yes	**	0.0035
FSam:0 d vs. FSol:60 d	-8667	-11259 to -6075	Yes	****	<0.0001

FSam:0 d vs. FSam+FSol:0 d	-5.457E-12	-2592 to 2592	No	ns	>0.9999
FSam:0 d vs. FSam+FSol:20 d	-2050	-4642 to 542.4	No	ns	0.8344
FSam:0 d vs. FSam+FSol:40 d	-10941	-13534 to -8349	Yes	****	<0.0001
FSam:0 d vs. FSam+FSol:60 d	-38056	-40648 to -35464	Yes	****	<0.0001
FSam:0 d vs. Control:0 d	-5.457E-12	-2592 to 2592	No	ns	>0.9999
FSam:0 d vs. Control:20 d	-1.819E-12	-2592 to 2592	No	ns	>0.9999
FSam:0 d vs. Control:40 d	-7.276E-12	-2592 to 2592	No	ns	>0.9999
FSam:0 d vs. Control:60 d	-5.457E-12	-2592 to 2592	No	ns	>0.9999
FSam:0 d vs. FSam:0 d	-2.728E-12	-2592 to 2592	No	ns	>0.9999
FSam:0 d vs. FSam:20 d	-719.2	-3311 to 1873	No	ns	>0.9999
FSam:0 d vs. FSam:40 d	-3745	-6337 to -1153	Yes	****	<0.0001
FSam:0 d vs. FSam:60 d	-8763	-11355 to -6171	Yes	****	<0.0001
FSam:0 d vs. FSol:0 d	-8.185E-12	-2592 to 2592	No	ns	>0.9999
FSam:0 d vs. FSol:20 d	-359.3	-2951 to 2233	No	ns	>0.9999
FSam:0 d vs. FSol:40 d	-1488	-4080 to 1104	No	ns	>0.9999
FSam:0 d vs. FSol:60 d	-4608	-7200 to -2016	Yes	****	<0.0001
FSam:0 d vs. FSam+FSol:0 d	-5.457E-12	-2592 to 2592	No	ns	>0.9999
FSam:0 d vs. FSam+FSol:20 d	-1061	-3653 to 1531	No	ns	>0.9999
FSam:0 d vs. FSam+FSol:40 d	-5606	-8198 to -3014	Yes	****	<0.0001
FSam:0 d vs. FSam+FSol:60 d	-19013	-21605 to -16421	Yes	****	<0.0001
FSam:20 d vs. FSam:40 d	-6139	-8731 to -3547	Yes	****	<0.0001
FSam:20 d vs. FSam:60 d	-27080	-29672 to -24488	Yes	****	<0.0001
FSam:20 d vs. FSol:0 d	1120	-1472 to 3712	No	ns	>0.9999
FSam:20 d vs. FSol:20 d	538.2	-2054 to 3130	No	ns	>0.9999
FSam:20 d vs. FSol:40 d	-1935	-4527 to 657.0	No	ns	>0.9999

FSam:20 d vs. FSol:60 d	-7547	-10139 to -4955	Yes	****	<0.0001
FSam:20 d vs. FSam+FSol:0 d	1120	-1472 to 3712	No	ns	>0.9999
FSam:20 d vs. FSam+FSol:20 d	-929.5	-3522 to 1663	No	ns	>0.9999
FSam:20 d vs. FSam+FSol:40 d	-9821	-12413 to -7229	Yes	****	<0.0001
FSam:20 d vs. FSam+FSol:60 d	-36936	-39528 to -34344	Yes	****	<0.0001
FSam:20 d vs. Control:0 d	1120	-1472 to 3712	No	ns	>0.9999
FSam:20 d vs. Control:20 d	1120	-1472 to 3712	No	ns	>0.9999
FSam:20 d vs. Control:40 d	1120	-1472 to 3712	No	ns	>0.9999
FSam:20 d vs. Control:60 d	1120	-1472 to 3712	No	ns	>0.9999
FSam:20 d vs. FSam:0 d	1120	-1472 to 3712	No	ns	>0.9999
FSam:20 d vs. FSam:20 d	400.9	-2191 to 2993	No	ns	>0.9999
FSam:20 d vs. FSam:40 d	-2625	-5217 to -32.89	Yes	*	0.0417
FSam:20 d vs. FSam:60 d	-7643	-10235 to -5051	Yes	****	<0.0001
FSam:20 d vs. FSol:0 d	1120	-1472 to 3712	No	ns	>0.9999
FSam:20 d vs. FSol:20 d	760.9	-1831 to 3353	No	ns	>0.9999
FSam:20 d vs. FSol:40 d	-368	-2960 to 2224	No	ns	>0.9999
FSam:20 d vs. FSol:60 d	-3488	-6080 to -896.2	Yes	***	0.0003
FSam:20 d vs. FSam+FSol:0 d	1120	-1472 to 3712	No	ns	>0.9999
FSam:20 d vs. FSam+FSol:20 d	59.47	-2533 to 2652	No	ns	>0.9999
FSam:20 d vs. FSam+FSol:40 d	-4486	-7078 to -1894	Yes	****	<0.0001
FSam:20 d vs. FSam+FSol:60 d	-17893	-20485 to -15301	Yes	****	<0.0001
FSam:40 d vs. FSam:60 d	-20942	-23534 to -18350	Yes	****	<0.0001
FSam:40 d vs. FSol:0 d	7259	4667 to 9851	Yes	****	<0.0001
FSam:40 d vs. FSol:20 d	6677	4085 to 9269	Yes	****	<0.0001
FSam:40 d vs. FSol:40 d	4204	1612 to 6796	Yes	****	<0.0001
FSam:40 d vs. FSol:60 d	-1408	-4000 to 1184	No	ns	>0.9999
FSam:40 d vs. FSam+FSol:0 d	7259	4667 to 9851	Yes	****	<0.0001
FSam:40 d vs. FSam+FSol:20 d	5209	2617 to 7801	Yes	****	<0.0001
FSam:40 d vs. FSam+FSol:40 d	-3683	-6275 to -1091	Yes	****	<0.0001
FSam:40 d vs. FSam+FSol:60 d	-30798	-33390 to -28205	Yes	****	<0.0001
FSam:40 d vs. Control:0 d	7259	4667 to 9851	Yes	****	<0.0001
FSam:40 d vs. Control:20 d	7259	4667 to 9851	Yes	****	<0.0001
FSam:40 d vs. Control:40 d	7259	4667 to 9851	Yes	****	<0.0001
FSam:40 d vs. Control:60 d	7259	4667 to 9851	Yes	****	<0.0001
FSam:40 d vs. FSam:0 d	7259	4667 to 9851	Yes	****	<0.0001
FSam:40 d vs. FSam:20 d	6540	3948 to 9132	Yes	****	<0.0001
FSam:40 d vs. FSam:40 d	3514	921.7 to 6106	Yes	***	0.0002
FSam:40 d vs. FSam:60 d	-1505	-4097 to 1088	No	ns	>0.9999

FSam:40 d vs. FSol:0 d	7259	4667 to 9851	Yes	****	<0.0001
FSam:40 d vs. FSol:20 d	6900	4308 to 9492	Yes	****	<0.0001
FSam:40 d vs. FSol:40 d	5771	3179 to 8363	Yes	****	<0.0001
FSam:40 d vs. FSol:60 d	2650	58.34 to 5242	Yes	*	0.0362
FSam:40 d vs. FSam+FSol:0 d	7259	4667 to 9851	Yes	****	<0.0001
FSam:40 d vs. FSam+FSol:20 d	6198	3606 to 8790	Yes	****	<0.0001
FSam:40 d vs. FSam+FSol:40 d	1653	-939.0 to 4245	No	ns	>0.9999
FSam:40 d vs. FSam+FSol:60 d	-11754	-14346 to -9162	Yes	****	<0.0001
FSam:60 d vs. FSol:0 d	28200	25608 to 30792	Yes	****	<0.0001
FSam:60 d vs. FSol:20 d	27618	25026 to 30210	Yes	****	<0.0001
FSam:60 d vs. FSol:40 d	25145	22553 to 27737	Yes	****	<0.0001
FSam:60 d vs. FSol:60 d	19534	16942 to 22126	Yes	****	<0.0001
FSam:60 d vs. FSam+FSol:0 d	28200	25608 to 30792	Yes	****	<0.0001
FSam:60 d vs. FSam+FSol:20 d	26151	23559 to 28743	Yes	****	<0.0001
FSam:60 d vs. FSam+FSol:40 d	17259	14667 to 19851	Yes	****	<0.0001
FSam:60 d vs. FSam+FSol:60 d	-9856	-12448 to -7264	Yes	****	<0.0001
FSam:60 d vs. Control:0 d	28200	25608 to 30792	Yes	****	<0.0001
FSam:60 d vs. Control:20 d	28200	25608 to 30792	Yes	****	<0.0001
FSam:60 d vs. Control:40 d	28200	25608 to 30792	Yes	****	<0.0001
FSam:60 d vs. Control:60 d	28200	25608 to 30792	Yes	****	<0.0001
FSam:60 d vs. FSam:0 d	28200	25608 to 30792	Yes	****	<0.0001
FSam:60 d vs. FSam:20 d	27481	24889 to 30073	Yes	****	<0.0001
FSam:60 d vs. FSam:40 d	24455	21863 to 27047	Yes	****	<0.0001
FSam:60 d vs. FSam:60 d	19437	16845 to 22029	Yes	****	<0.0001
FSam:60 d vs. FSol:0 d	28200	25608 to 30792	Yes	****	<0.0001
FSam:60 d vs. FSol:20 d	27841	25249 to 30433	Yes	****	<0.0001
FSam:60 d vs. FSol:40 d	26712	24120 to 29304	Yes	****	<0.0001
FSam:60 d vs. FSol:60 d	23592	21000 to 26184	Yes	****	<0.0001
FSam:60 d vs. FSam+FSol:0 d	28200	25608 to 30792	Yes	****	<0.0001

FSam:60 d vs. FSam+FSol:20 d	27140	24548 to 29732	Yes	****	<0.0001
FSam:60 d vs. FSam+FSol:40 d	22595	20003 to 25187	Yes	****	<0.0001
FSam:60 d vs. FSam+FSol:60 d	9187	6595 to 11779	Yes	****	<0.0001
FSol:0 d vs. FSol:20 d	-581.9	-3174 to 2010	No	ns	>0.9999
FSol:0 d vs. FSol:40 d	-3055	-5647 to -463.2	Yes	**	0.0035
FSol:0 d vs. FSol:60 d	-8667	-11259 to -6075	Yes	****	<0.0001
FSol:0 d vs. FSam+FSol:0 d	0	-2592 to 2592	No	ns	>0.9999
FSol:0 d vs. FSam+FSol:20 d	-2050	-4642 to 542.4	No	ns	0.8344
FSol:0 d vs. FSam+FSol:40 d	-10941	-13534 to -8349	Yes	****	<0.0001
FSol:0 d vs. FSam+FSol:60 d	-38056	-40648 to -35464	Yes	****	<0.0001
FSol:0 d vs. Control:0 d	0	-2592 to 2592	No	ns	>0.9999
FSol:0 d vs. Control:20 d	3.638E-12	-2592 to 2592	No	ns	>0.9999
FSol:0 d vs. Control:40 d	-1.819E-12	-2592 to 2592	No	ns	>0.9999
FSol:0 d vs. Control:60 d	0	-2592 to 2592	No	ns	>0.9999
FSol:0 d vs. FSam:0 d	2.728E-12	-2592 to 2592	No	ns	>0.9999
FSol:0 d vs. FSam:20 d	-719.2	-3311 to 1873	No	ns	>0.9999
FSol:0 d vs. FSam:40 d	-3745	-6337 to -1153	Yes	****	<0.0001
FSol:0 d vs. FSam:60 d	-8763	-11355 to -6171	Yes	****	<0.0001
FSol:0 d vs. FSol:0 d	-2.728E-12	-2592 to 2592	No	ns	>0.9999
FSol:0 d vs. FSol:20 d	-359.3	-2951 to 2233	No	ns	>0.9999
FSol:0 d vs. FSol:40 d	-1488	-4080 to 1104	No	ns	>0.9999
FSol:0 d vs. FSol:60 d	-4608	-7200 to -2016	Yes	****	<0.0001
FSol:0 d vs. FSam+FSol:0 d	0	-2592 to 2592	No	ns	>0.9999
FSol:0 d vs. FSam+FSol:20 d	-1061	-3653 to 1531	No	ns	>0.9999
FSol:0 d vs. FSam+FSol:40 d	-5606	-8198 to -3014	Yes	****	<0.0001
FSol:0 d vs. FSam+FSol:60 d	-19013	-21605 to -16421	Yes	****	<0.0001
FSol:20 d vs. FSol:40 d	-2473	-5065 to 118.7	No	ns	0.0954
FSol:20 d vs. FSol:60 d	-8085	-10677 to -5493	Yes	****	<0.0001
FSol:20 d vs. FSam+FSol:0 d	581.9	-2010 to 3174	No	ns	>0.9999
FSol:20 d vs. FSam+FSol:20 d	-1468	-4060 to 1124	No	ns	>0.9999
FSol:20 d vs. FSam+FSol:40 d	-10360	-12952 to -7767	Yes	****	<0.0001

FSol:20 d vs. FSam+FSol:60 d	-37474	-40066 to -34882	Yes	****	<0.0001
FSol:20 d vs. Control:0 d	581.9	-2010 to 3174	No	ns	>0.9999
FSol:20 d vs. Control:20 d	581.9	-2010 to 3174	No	ns	>0.9999
FSol:20 d vs. Control:40 d	581.9	-2010 to 3174	No	ns	>0.9999
FSol:20 d vs. Control:60 d	581.9	-2010 to 3174	No	ns	>0.9999
FSol:20 d vs. FSam:0 d	581.9	-2010 to 3174	No	ns	>0.9999
FSol:20 d vs. FSam:20 d	-137.3	-2729 to 2455	No	ns	>0.9999
FSol:20 d vs. FSam:40 d	-3163	-5755 to -571.1	Yes	**	0.0019
FSol:20 d vs. FSam:60 d	-8181	-10773 to -5589	Yes	****	<0.0001
FSol:20 d vs. FSol:0 d	581.9	-2010 to 3174	No	ns	>0.9999
FSol:20 d vs. FSol:20 d	222.7	-2369 to 2815	No	ns	>0.9999
FSol:20 d vs. FSol:40 d	-906.2	-3498 to 1686	No	ns	>0.9999
FSol:20 d vs. FSol:60 d	-4026	-6619 to -1434	Yes	****	<0.0001
FSol:20 d vs. FSam+FSol:0 d	581.9	-2010 to 3174	No	ns	>0.9999
FSol:20 d vs. FSam+FSol:20 d	-478.8	-3071 to 2113	No	ns	>0.9999
FSol:20 d vs. FSam+FSol:40 d	-5024	-7616 to -2432	Yes	****	<0.0001
FSol:20 d vs. FSam+FSol:60 d	-18431	-21023 to -15839	Yes	****	<0.0001
FSol:40 d vs. FSol:60 d	-5612	-8204 to -3020	Yes	****	<0.0001
FSol:40 d vs. FSam+FSol:0 d	3055	463.2 to 5647	Yes	**	0.0035
FSol:40 d vs. FSam+FSol:20 d	1006	-1586 to 3598	No	ns	>0.9999
FSol:40 d vs. FSam+FSol:40 d	-7886	-10478 to -5294	Yes	****	<0.0001
FSol:40 d vs. FSam+FSol:60 d	-35001	-37593 to -32409	Yes	****	<0.0001
FSol:40 d vs. Control:0 d	3055	463.2 to 5647	Yes	**	0.0035
FSol:40 d vs. Control:20 d	3055	463.2 to 5647	Yes	**	0.0035
FSol:40 d vs. Control:40 d	3055	463.2 to 5647	Yes	**	0.0035
FSol:40 d vs. Control:60 d	3055	463.2 to 5647	Yes	**	0.0035
FSol:40 d vs. FSam:0 d	3055	463.2 to 5647	Yes	**	0.0035
FSol:40 d vs. FSam:20 d	2336	-256.1 to 4928	No	ns	0.1973
FSol:40 d vs. FSam:40 d	-689.9	-3282 to 1902	No	ns	>0.9999
FSol:40 d vs. FSam:60 d	-5708	-8300 to -3116	Yes	****	<0.0001
FSol:40 d vs. FSol:0 d	3055	463.2 to 5647	Yes	**	0.0035
FSol:40 d vs. FSol:20 d	2696	103.9 to 5288	Yes	*	0.0281
FSol:40 d vs. FSol:40 d	1567	-1025 to 4159	No	ns	>0.9999
FSol:40 d vs. FSol:60 d	-1553	-4145 to 1039	No	ns	>0.9999
FSol:40 d vs. FSam+FSol:0 d	3055	463.2 to 5647	Yes	**	0.0035
FSol:40 d vs. FSam+FSol:20 d	1995	-597.5 to 4587	No	ns	>0.9999

FSol:40 d vs. FSam+FSol:40 d	-2551	-5143 to 41.53	No	ns	0.0628
FSol:40 d vs. FSam+FSol:60 d	-15958	-18550 to -13366	Yes	****	<0.0001
FSol:60 d vs. FSam+FSol:0 d	8667	6075 to 11259	Yes	****	<0.0001
FSol:60 d vs. FSam+FSol:20 d	6617	4025 to 9209	Yes	****	<0.0001
FSol:60 d vs. FSam+FSol:40 d	-2275	-4867 to 317.4	No	ns	0.2711
FSol:60 d vs. FSam+FSol:60 d	-29390	-31982 to -26798	Yes	****	<0.0001
FSol:60 d vs. Control:0 d	8667	6075 to 11259	Yes	****	<0.0001
FSol:60 d vs. Control:20 d	8667	6075 to 11259	Yes	****	<0.0001
FSol:60 d vs. Control:40 d	8667	6075 to 11259	Yes	****	<0.0001
FSol:60 d vs. Control:60 d	8667	6075 to 11259	Yes	****	<0.0001
FSol:60 d vs. FSam:0 d	8667	6075 to 11259	Yes	****	<0.0001
FSol:60 d vs. FSam:20 d	7948	5356 to 10540	Yes	****	<0.0001
FSol:60 d vs. FSam:40 d	4922	2330 to 7514	Yes	****	<0.0001
FSol:60 d vs. FSam:60 d	-96.56	-2689 to 2495	No	ns	>0.9999
FSol:60 d vs. FSol:0 d	8667	6075 to 11259	Yes	****	<0.0001
FSol:60 d vs. FSol:20 d	8308	5716 to 10900	Yes	****	<0.0001
FSol:60 d vs. FSol:40 d	7179	4587 to 9771	Yes	****	<0.0001
FSol:60 d vs. FSol:60 d	4058	1466 to 6650	Yes	****	<0.0001
FSol:60 d vs. FSam+FSol:0 d	8667	6075 to 11259	Yes	****	<0.0001
FSol:60 d vs. FSam+FSol:20 d	7606	5014 to 10198	Yes	****	<0.0001
FSol:60 d vs. FSam+FSol:40 d	3061	469.0 to 5653	Yes	**	0.0034
FSol:60 d vs. FSam+FSol:60 d	-10346	-12938 to -7754	Yes	****	<0.0001
FSam+FSol:0 d vs. FSam+FSol:20 d	-2050	-4642 to 542.4	No	ns	0.8344
FSam+FSol:0 d vs. FSam+FSol:40 d	-10941	-13534 to -8349	Yes	****	<0.0001
FSam+FSol:0 d vs. FSam+FSol:60 d	-38056	-40648 to -35464	Yes	****	<0.0001
FSam+FSol:0 d vs. Control:0 d	0	-2592 to 2592	No	ns	>0.9999
FSam+FSol:0 d vs. Control:20 d	3.638E-12	-2592 to 2592	No	ns	>0.9999
FSam+FSol:0 d vs. Control:40 d	-1.819E-12	-2592 to 2592	No	ns	>0.9999
FSam+FSol:0 d vs. Control:60 d	0	-2592 to 2592	No	ns	>0.9999
FSam+FSol:0 d vs. FSam:0 d	2.728E-12	-2592 to 2592	No	ns	>0.9999
FSam+FSol:0 d vs. FSam:20 d	-719.2	-3311 to 1873	No	ns	>0.9999
FSam+FSol:0 d vs. FSam:40 d	-3745	-6337 to -1153	Yes	****	<0.0001
FSam+FSol:0 d vs. FSam:60 d	-8763	-11355 to -6171	Yes	****	<0.0001
FSam+FSol:0 d vs. FSol:0 d	-2.728E-12	-2592 to 2592	No	ns	>0.9999

FSam+FSol:0 d vs. FSol:20 d	-359.3	-2951 to 2233	No	ns	>0.9999
FSam+FSol:0 d vs. FSol:40 d	-1488	-4080 to 1104	No	ns	>0.9999
FSam+FSol:0 d vs. FSol:60 d	-4608	-7200 to -2016	Yes	****	<0.0001
FSam+FSol:0 d vs. FSam+FSol:0 d	0	-2592 to 2592	No	ns	>0.9999
FSam+FSol:0 d vs. FSam+FSol:20 d	-1061	-3653 to 1531	No	ns	>0.9999
FSam+FSol:0 d vs. FSam+FSol:40 d	-5606	-8198 to -3014	Yes	****	<0.0001
FSam+FSol:0 d vs. FSam+FSol:60 d	-19013	-21605 to -16421	Yes	****	<0.0001
FSam+FSol:20 d vs. FSam+FSol:40 d	-8892	-11484 to -6300	Yes	****	<0.0001
FSam+FSol:20 d vs. FSam+FSol:60 d	-36007	-38599 to -33415	Yes	****	<0.0001
FSam+FSol:20 d vs. Control:0 d	2050	-542.4 to 4642	No	ns	0.8344
FSam+FSol:20 d vs. Control:20 d	2050	-542.4 to 4642	No	ns	0.8344
FSam+FSol:20 d vs. Control:40 d	2050	-542.4 to 4642	No	ns	0.8344
FSam+FSol:20 d vs. Control:60 d	2050	-542.4 to 4642	No	ns	0.8344
FSam+FSol:20 d vs. FSam:0 d	2050	-542.4 to 4642	No	ns	0.8344
FSam+FSol:20 d vs. FSam:20 d	1330	-1262 to 3922	No	ns	>0.9999
FSam+FSol:20 d vs. FSam:40 d	-1695	-4287 to 896.6	No	ns	>0.9999
FSam+FSol:20 d vs. FSam:60 d	-6714	-9306 to -4122	Yes	****	<0.0001
FSam+FSol:20 d vs. FSol:0 d	2050	-542.4 to 4642	No	ns	0.8344
FSam+FSol:20 d vs. FSol:20 d	1690	-901.6 to 4282	No	ns	>0.9999
FSam+FSol:20 d vs. FSol:40 d	561.5	-2031 to 3154	No	ns	>0.9999
FSam+FSol:20 d vs. FSol:60 d	-2559	-5151 to 33.30	No	ns	0.06
FSam+FSol:20 d vs. FSam+FSol:0 d	2050	-542.4 to 4642	No	ns	0.8344
FSam+FSol:20 d vs. FSam+FSol:20 d	989	-1603 to 3581	No	ns	>0.9999
FSam+FSol:20 d vs. FSam+FSol:40 d	-3556	-6148 to -964.0	Yes	***	0.0002
FSam+FSol:20 d vs. FSam+FSol:60 d	-16963	-19555 to -14371	Yes	****	<0.0001
FSam+FSol:40 d vs. FSam+FSol:60 d	-27115	-29707 to -24523	Yes	****	<0.0001
FSam+FSol:40 d vs. Control:0 d	10941	8349 to 13534	Yes	****	<0.0001
FSam+FSol:40 d vs. Control:20 d	10941	8349 to 13534	Yes	****	<0.0001
FSam+FSol:40 d vs. Control:40 d	10941	8349 to 13534	Yes	****	<0.0001
FSam+FSol:40 d vs. Control:60 d	10941	8349 to 13534	Yes	****	<0.0001

FSam+FSol:40 d vs. FSam:0 d	10941	8349 to 13534	Yes	****	<0.0001
FSam+FSol:40 d vs. FSam:20 d	10222	7630 to 12814	Yes	****	<0.0001
FSam+FSol:40 d vs. FSam:40 d	7196	4604 to 9788	Yes	****	<0.0001
FSam+FSol:40 d vs. FSam:60 d	2178	-414.0 to 4770	No	ns	0.4428
FSam+FSol:40 d vs. FSol:0 d	10941	8349 to 13534	Yes	****	<0.0001
FSam+FSol:40 d vs. FSol:20 d	10582	7990 to 13174	Yes	****	<0.0001
FSam+FSol:40 d vs. FSol:40 d	9453	6861 to 12045	Yes	****	<0.0001
FSam+FSol:40 d vs. FSol:60 d	6333	3741 to 8925	Yes	****	<0.0001
FSam+FSol:40 d vs. FSam+FSol:0 d	10941	8349 to 13534	Yes	****	<0.0001
FSam+FSol:40 d vs. FSam+FSol:20 d	9881	7289 to 12473	Yes	****	<0.0001
FSam+FSol:40 d vs. FSam+FSol:40 d	5336	2744 to 7928	Yes	****	<0.0001
FSam+FSol:40 d vs. FSam+FSol:60 d	-8072	-10664 to -5480	Yes	****	<0.0001
FSam+FSol:60 d vs. Control:0 d	38056	35464 to 40648	Yes	****	<0.0001
FSam+FSol:60 d vs. Control:20 d	38056	35464 to 40648	Yes	****	<0.0001
FSam+FSol:60 d vs. Control:40 d	38056	35464 to 40648	Yes	****	<0.0001
FSam+FSol:60 d vs. Control:60 d	38056	35464 to 40648	Yes	****	<0.0001
FSam+FSol:60 d vs. FSam:0 d	38056	35464 to 40648	Yes	****	<0.0001
FSam+FSol:60 d vs. FSam:20 d	37337	34745 to 39929	Yes	****	<0.0001
FSam+FSol:60 d vs. FSam:40 d	34311	31719 to 36903	Yes	****	<0.0001
FSam+FSol:60 d vs. FSam:60 d	29293	26701 to 31885	Yes	****	<0.0001
FSam+FSol:60 d vs. FSol:0 d	38056	35464 to 40648	Yes	****	<0.0001
FSam+FSol:60 d vs. FSol:20 d	37697	35105 to 40289	Yes	****	<0.0001
FSam+FSol:60 d vs. FSol:40 d	36568	33976 to 39160	Yes	****	<0.0001
FSam+FSol:60 d vs. FSol:60 d	33448	30856 to 36040	Yes	****	<0.0001
FSam+FSol:60 d vs. FSam+FSol:0 d	38056	35464 to 40648	Yes	****	<0.0001
FSam+FSol:60 d vs. FSam+FSol:20 d	36996	34404 to 39588	Yes	****	<0.0001
FSam+FSol:60 d vs. FSam+FSol:40 d	32451	29859 to 35043	Yes	****	<0.0001
FSam+FSol:60 d vs. FSam+FSol:60 d	19043	16451 to 21635	Yes	****	<0.0001
Control:0 d vs. Control:20 d	3.638E-12	-2592 to 2592	No	ns	>0.9999

Control:0 d vs. Control:40 d	-1.819E-12	-2592 to 2592	No	ns	>0.9999
Control:0 d vs. Control:60 d	0	-2592 to 2592	No	ns	>0.9999
Control:0 d vs. FSam:0 d	2.728E-12	-2592 to 2592	No	ns	>0.9999
Control:0 d vs. FSam:20 d	-719.2	-3311 to 1873	No	ns	>0.9999
Control:0 d vs. FSam:40 d	-3745	-6337 to -1153	Yes	****	<0.0001
Control:0 d vs. FSam:60 d	-8763	-11355 to -6171	Yes	****	<0.0001
Control:0 d vs. FSol:0 d	-2.728E-12	-2592 to 2592	No	ns	>0.9999
Control:0 d vs. FSol:20 d	-359.3	-2951 to 2233	No	ns	>0.9999
Control:0 d vs. FSol:40 d	-1488	-4080 to 1104	No	ns	>0.9999
Control:0 d vs. FSol:60 d	-4608	-7200 to -2016	Yes	****	<0.0001
Control:0 d vs. FSam+FSol:0 d	0	-2592 to 2592	No	ns	>0.9999
Control:0 d vs. FSam+FSol:20 d	-1061	-3653 to 1531	No	ns	>0.9999
Control:0 d vs. FSam+FSol:40 d	-5606	-8198 to -3014	Yes	****	<0.0001
Control:0 d vs. FSam+FSol:60 d	-19013	-21605 to -16421	Yes	****	<0.0001
Control:20 d vs. Control:40 d	-5.457E-12	-2592 to 2592	No	ns	>0.9999
Control:20 d vs. Control:60 d	-3.638E-12	-2592 to 2592	No	ns	>0.9999
Control:20 d vs. FSam:0 d	-9.095E-13	-2592 to 2592	No	ns	>0.9999
Control:20 d vs. FSam:20 d	-719.2	-3311 to 1873	No	ns	>0.9999
Control:20 d vs. FSam:40 d	-3745	-6337 to -1153	Yes	****	<0.0001
Control:20 d vs. FSam:60 d	-8763	-11355 to -6171	Yes	****	<0.0001
Control:20 d vs. FSol:0 d	-6.366E-12	-2592 to 2592	No	ns	>0.9999
Control:20 d vs. FSol:20 d	-359.3	-2951 to 2233	No	ns	>0.9999
Control:20 d vs. FSol:40 d	-1488	-4080 to 1104	No	ns	>0.9999
Control:20 d vs. FSol:60 d	-4608	-7200 to -2016	Yes	****	<0.0001
Control:20 d vs. FSam+FSol:0 d	-3.638E-12	-2592 to 2592	No	ns	>0.9999
Control:20 d vs. FSam+FSol:20 d	-1061	-3653 to 1531	No	ns	>0.9999
Control:20 d vs. FSam+FSol:40 d	-5606	-8198 to -3014	Yes	****	<0.0001

Control:20 d vs. FSam+FSol:60 d	-19013	-21605 to -16421	Yes	****	<0.0001
Control:40 d vs. Control:60 d	1.819E-12	-2592 to 2592	No	ns	>0.9999
Control:40 d vs. FSam:0 d	4.547E-12	-2592 to 2592	No	ns	>0.9999
Control:40 d vs. FSam:20 d	-719.2	-3311 to 1873	No	ns	>0.9999
Control:40 d vs. FSam:40 d	-3745	-6337 to -1153	Yes	****	<0.0001
Control:40 d vs. FSam:60 d	-8763	-11355 to -6171	Yes	****	<0.0001
Control:40 d vs. FSol:0 d	-9.095E-13	-2592 to 2592	No	ns	>0.9999
Control:40 d vs. FSol:20 d	-359.3	-2951 to 2233	No	ns	>0.9999
Control:40 d vs. FSol:40 d	-1488	-4080 to 1104	No	ns	>0.9999
Control:40 d vs. FSol:60 d	-4608	-7200 to -2016	Yes	****	<0.0001
Control:40 d vs. FSam+FSol:0 d	1.819E-12	-2592 to 2592	No	ns	>0.9999
Control:40 d vs. FSam+FSol:20 d	-1061	-3653 to 1531	No	ns	>0.9999
Control:40 d vs. FSam+FSol:40 d	-5606	-8198 to -3014	Yes	****	<0.0001
Control:40 d vs. FSam+FSol:60 d	-19013	-21605 to -16421	Yes	****	<0.0001
Control:60 d vs. FSam:0 d	2.728E-12	-2592 to 2592	No	ns	>0.9999
Control:60 d vs. FSam:20 d	-719.2	-3311 to 1873	No	ns	>0.9999
Control:60 d vs. FSam:40 d	-3745	-6337 to -1153	Yes	****	<0.0001
Control:60 d vs. FSam:60 d	-8763	-11355 to -6171	Yes	****	<0.0001
Control:60 d vs. FSol:0 d	-2.728E-12	-2592 to 2592	No	ns	>0.9999
Control:60 d vs. FSol:20 d	-359.3	-2951 to 2233	No	ns	>0.9999
Control:60 d vs. FSol:40 d	-1488	-4080 to 1104	No	ns	>0.9999
Control:60 d vs. FSol:60 d	-4608	-7200 to -2016	Yes	****	<0.0001
Control:60 d vs. FSam+FSol:0 d	0	-2592 to 2592	No	ns	>0.9999
Control:60 d vs. FSam+FSol:20 d	-1061	-3653 to 1531	No	ns	>0.9999
Control:60 d vs. FSam+FSol:40 d	-5606	-8198 to -3014	Yes	****	<0.0001
Control:60 d vs. FSam+FSol:60 d	-19013	-21605 to -16421	Yes	****	<0.0001
FSam:0 d vs. FSam:20 d	-719.2	-3311 to 1873	No	ns	>0.9999
FSam:0 d vs. FSam:40 d	-3745	-6337 to -1153	Yes	****	<0.0001
FSam:0 d vs. FSam:60 d	-8763	-11355 to -6171	Yes	****	<0.0001

FSam:0 d vs. FSol:0 d	-5.457E-12	-2592 to 2592	No	ns	>0.9999
FSam:0 d vs. FSol:20 d	-359.3	-2951 to 2233	No	ns	>0.9999
FSam:0 d vs. FSol:40 d	-1488	-4080 to 1104	No	ns	>0.9999
FSam:0 d vs. FSol:60 d	-4608	-7200 to -2016	Yes	****	<0.0001
FSam:0 d vs. FSam+FSol:0 d	-2.728E-12	-2592 to 2592	No	ns	>0.9999
FSam:0 d vs. FSam+FSol:20 d	-1061	-3653 to 1531	No	ns	>0.9999
FSam:0 d vs. FSam+FSol:40 d	-5606	-8198 to -3014	Yes	****	<0.0001
FSam:0 d vs. FSam+FSol:60 d	-19013	-21605 to -16421	Yes	****	<0.0001
FSam:20 d vs. FSam:40 d	-3026	-5618 to -433.8	Yes	**	0.0042
FSam:20 d vs. FSam:60 d	-8044	-10636 to -5452	Yes	****	<0.0001
FSam:20 d vs. FSol:0 d	719.2	-1873 to 3311	No	ns	>0.9999
FSam:20 d vs. FSol:20 d	360	-2232 to 2952	No	ns	>0.9999
FSam:20 d vs. FSol:40 d	-768.9	-3361 to 1823	No	ns	>0.9999
FSam:20 d vs. FSol:60 d	-3889	-6481 to -1297	Yes	****	<0.0001
FSam:20 d vs. FSam+FSol:0 d	719.2	-1873 to 3311	No	ns	>0.9999
FSam:20 d vs. FSam+FSol:20 d	-341.5	-2934 to 2251	No	ns	>0.9999
FSam:20 d vs. FSam+FSol:40 d	-4887	-7479 to -2294	Yes	****	<0.0001
FSam:20 d vs. FSam+FSol:60 d	-18294	-20886 to -15702	Yes	****	<0.0001
FSam:40 d vs. FSam:60 d	-5018	-7610 to -2426	Yes	****	<0.0001
FSam:40 d vs. FSol:0 d	3745	1153 to 6337	Yes	****	<0.0001
FSam:40 d vs. FSol:20 d	3386	793.8 to 5978	Yes	***	0.0005
FSam:40 d vs. FSol:40 d	2257	-335.1 to 4849	No	ns	0.2969
FSam:40 d vs. FSol:60 d	-863.3	-3455 to 1729	No	ns	>0.9999
FSam:40 d vs. FSam+FSol:0 d	3745	1153 to 6337	Yes	****	<0.0001
FSam:40 d vs. FSam+FSol:20 d	2684	92.36 to 5276	Yes	*	0.03
FSam:40 d vs. FSam+FSol:40 d	-1861	-4453 to 731.4	No	ns	>0.9999
FSam:40 d vs. FSam+FSol:60 d	-15268	-17860 to -12676	Yes	****	<0.0001
FSam:60 d vs. FSol:0 d	8763	6171 to 11355	Yes	****	<0.0001
FSam:60 d vs. FSol:20 d	8404	5812 to 10996	Yes	****	<0.0001
FSam:60 d vs. FSol:40 d	7275	4683 to 9867	Yes	****	<0.0001
FSam:60 d vs. FSol:60 d	4155	1563 to 6747	Yes	****	<0.0001
FSam:60 d vs. FSam+FSol:0 d	8763	6171 to 11355	Yes	****	<0.0001
FSam:60 d vs. FSam+FSol:20 d	7703	5111 to 10295	Yes	****	<0.0001
FSam:60 d vs. FSam+FSol:40 d	3158	565.5 to 5750	Yes	**	0.0019

FSam:60 d vs. FSam+FSol:60 d	-10250	-12842 to -7658	Yes	****	<0.0001
FSol:0 d vs. FSol:20 d	-359.3	-2951 to 2233	No	ns	>0.9999
FSol:0 d vs. FSol:40 d	-1488	-4080 to 1104	No	ns	>0.9999
FSol:0 d vs. FSol:60 d	-4608	-7200 to -2016	Yes	****	<0.0001
FSol:0 d vs. FSam+FSol:0 d	2.728E-12	-2592 to 2592	No	ns	>0.9999
FSol:0 d vs. FSam+FSol:20 d	-1061	-3653 to 1531	No	ns	>0.9999
FSol:0 d vs. FSam+FSol:40 d	-5606	-8198 to -3014	Yes	****	<0.0001
FSol:0 d vs. FSam+FSol:60 d	-19013	-21605 to -16421	Yes	****	<0.0001
FSol:20 d vs. FSol:40 d	-1129	-3721 to 1463	No	ns	>0.9999
FSol:20 d vs. FSol:60 d	-4249	-6841 to -1657	Yes	****	<0.0001
FSol:20 d vs. FSam+FSol:0 d	359.3	-2233 to 2951	No	ns	>0.9999
FSol:20 d vs. FSam+FSol:20 d	-701.4	-3293 to 1891	No	ns	>0.9999
FSol:20 d vs. FSam+FSol:40 d	-5247	-7839 to -2654	Yes	****	<0.0001
FSol:20 d vs. FSam+FSol:60 d	-18654	-21246 to -16062	Yes	****	<0.0001
FSol:40 d vs. FSol:60 d	-3120	-5712 to -528.2	Yes	**	0.0024
FSol:40 d vs. FSam+FSol:0 d	1488	-1104 to 4080	No	ns	>0.9999
FSol:40 d vs. FSam+FSol:20 d	427.5	-2165 to 3020	No	ns	>0.9999
FSol:40 d vs. FSam+FSol:40 d	-4118	-6710 to -1526	Yes	****	<0.0001
FSol:40 d vs. FSam+FSol:60 d	-17525	-20117 to -14933	Yes	****	<0.0001
FSol:60 d vs. FSam+FSol:0 d	4608	2016 to 7200	Yes	****	<0.0001
FSol:60 d vs. FSam+FSol:20 d	3548	955.7 to 6140	Yes	***	0.0002
FSol:60 d vs. FSam+FSol:40 d	-997.3	-3589 to 1595	No	ns	>0.9999
FSol:60 d vs. FSam+FSol:60 d	-14405	-16997 to -11813	Yes	****	<0.0001
FSam+FSol:0 d vs. FSam+FSol:20 d	-1061	-3653 to 1531	No	ns	>0.9999
FSam+FSol:0 d vs. FSam+FSol:40 d	-5606	-8198 to -3014	Yes	****	<0.0001
FSam+FSol:0 d vs. FSam+FSol:60 d	-19013	-21605 to -16421	Yes	****	<0.0001
FSam+FSol:20 d vs. FSam+FSol:40 d	-4545	-7137 to -1953	Yes	****	<0.0001
FSam+FSol:20 d vs. FSam+FSol:60 d	-17952	-20544 to -15360	Yes	****	<0.0001
FSam+FSol:40 d vs. FSam+FSol:60 d	-13407	-15999 to -10815	Yes	****	<0.0001

Table S6: Tukey multiple comparison test for starch content of Kufri Jyoti and Kufri Frysona when infected with control (water agar), *Fusarium Sambucinum, Fusarium solani* and *Fusarium Sambucinum + Fusarium solani* and incubated for 0 d, 20 d, 40 d and 60 days

Tukey's multiple comparisons test	Significant?	Summary	Adjusted P Value
control			
0 d vs. 20 d	No	ns	0.9644
0 d vs. 40 d	No	ns	0.9948
0 d vs. 60 d	No	ns	0.3806
20 d vs. 40 d	No	ns	0.9957
20 d vs. 60 d	No	ns	0.1692
40 d vs. 60 d	No	ns	0.2577
Fsam			
0 d vs. 20 d	No	ns	0.9968
0 d vs. 40 d	Yes	***	0.0002
0 d vs. 60 d	Yes	****	<0.0001
20 d vs. 40 d	Yes	***	0.0004
20 d vs. 60 d	Yes	****	<0.0001
40 d vs. 60 d	Yes	****	<0.0001
Fsol			
0 d vs. 20 d	No	ns	0.6094
0 d vs. 40 d	No	ns	0.1843
0 d vs. 60 d	Yes	*	0.0126
20 d vs. 40 d	Yes	**	0.0091
20 d vs. 60 d	Yes	***	0.0002
40 d vs. 60 d	No	ns	0.6794
FSam+FSol			
0 d vs. 20 d	No	ns	0.948
0 d vs. 40 d	Yes	*	0.0197
0 d vs. 60 d	Yes	****	<0.0001
20 d vs. 40 d	Yes	**	0.0041
20 d vs. 60 d	Yes	****	<0.0001
40 d vs. 60 d	Yes	****	<0.0001
control			
0 d vs. 20 d	No	ns	0.9013
0 d vs. 40 d	No	ns	0.2524
0 d vs. 60 d	Yes	**	0.0048
20 d vs. 40 d	No	ns	0.6422
20 d vs. 60 d	Yes	*	0.0336
40 d vs. 60 d	No	ns	0.3761
Fsam			
0 d vs. 20 d	Yes	*	0.0138
0 d vs. 40 d	Yes	****	<0.0001
0 d vs. 60 d	Yes	****	<0.0001

20 d vs. 40 d	Yes	****	<0.0001
20 d vs. 60 d	Yes	****	<0.0001
40 d vs. 60 d	Yes	***	0.0007
Fsol			
0 d vs. 20 d	Yes	****	<0.0001
0 d vs. 40 d	Yes	****	<0.0001
0 d vs. 60 d	Yes	****	<0.0001
20 d vs. 40 d	Yes	***	0.001
20 d vs. 60 d	Yes	****	<0.0001
40 d vs. 60 d	No	ns	0.1407
FSam+FSol			
0 d vs. 20 d	Yes	****	<0.0001
0 d vs. 40 d	Yes	****	<0.0001
0 d vs. 60 d	Yes	****	<0.0001
20 d vs. 40 d	No	ns	0.0716
20 d vs. 60 d	Yes	****	<0.0001
40 d vs. 60 d	Yes	***	0.0004

Table S7: Tukey multiple comparison test for Glycemic index of Kufri Jyoti and Kufri Frysona when infected with control (water agar), *Fusarium Sambucinum*, *Fusarium solani* and *Fusarium Sambucinum* + *Fusarium solani* and incubated for 0 d, 20 d, 40 d and 60 days

Tukey's multiple comparisons test	Mean Diff.	95.00% CI of diff.	Significant?	Summary	Adjusted P Value
0 d					
Control vs. FSam	-2.533	-3.645 to -1.421	Yes	***	<.001
Control vs. FSol	-1.393	-2.505 to -0.2814	Yes	**	0.005
Control vs. FSam+FSol	-2.717	-3.829 to -1.605	Yes	***	<.001
Control vs. Control	-7.413	-8.525 to -6.301	Yes	***	<.001
Control vs. FSam	-8.817	-9.929 to -7.705	Yes	***	<.001
Control vs. FSol	-7.893	-9.005 to -6.781	Yes	***	<.001
Control vs. FSam+FSol	-9.813	-10.93 to -8.701	Yes	***	<.001
FSam vs. FSol	1.14	0.02803 to 2.252	Yes	*	0.041
FSam vs. FSam+FSol	-0.1833	-1.295 to 0.9286	No	ns	>.999
FSam vs. Control	-4.88	-5.992 to -3.768	Yes	***	<.001
FSam vs. FSam	-6.283	-7.395 to -5.171	Yes	***	<.001
FSam vs. FSol	-5.36	-6.472 to -4.248	Yes	***	<.001
FSam vs. FSam+FSol	-7.28	-8.392 to -6.168	Yes	***	<.001
FSol vs. FSam+FSol	-1.323	-2.435 to -0.2114	Yes	**	0.009
FSol vs. Control	-6.02	-7.132 to -4.908	Yes	***	<.001

FSol vs. FSam	-7.423	-8.535 to -6.311	Yes	***	<.001
FSol vs. FSol	-6.5	-7.612 to -5.388	Yes	***	<.001
FSol vs. FSam+FSol	-8.42	-9.532 to -7.308	Yes	***	<.001
FSam+FSol vs. Control	-4.697	-5.809 to -3.585	Yes	***	<.001
FSam+FSol vs. FSam	-6.1	-7.212 to -4.988	Yes	***	<.001
FSam+FSol vs. FSol	-5.177	-6.289 to -4.065	Yes	***	<.001
FSam+FSol vs. FSam+FSol	-7.097	-8.209 to -5.985	Yes	***	<.001
Control vs. FSam	-1.403	-2.515 to -0.2914	Yes	**	0.005
Control vs. FSol	-0.48	-1.592 to 0.6320	No	ns	0.875
Control vs. FSam+FSol	-2.4	-3.512 to -1.288	Yes	***	<.001
FSam vs. FSol	0.9233	-0.1886 to 2.035	No	ns	0.174
FSam vs. FSam+FSol	-0.9967	-2.109 to 0.1153	No	ns	0.111
FSol vs. FSam+FSol	-1.92	-3.032 to -0.8080	Yes	***	<.001
20 d					
Control vs. FSam	-2.807	-3.919 to -1.695	Yes	***	<.001
Control vs. FSol	-1.687	-2.799 to -0.5747	Yes	***	<.001
Control vs. FSam+FSol	-3.687	-4.799 to -2.575	Yes	***	<.001
Control vs. Control	-7.143	-8.255 to -6.031	Yes	***	<.001
Control vs. FSam	-8.92	-10.03 to -7.808	Yes	***	<.001
Control vs. FSol	-8.157	-9.269 to -7.045	Yes	***	<.001
Control vs. FSam+FSol	-10.94	-12.05 to -9.825	Yes	***	<.001
FSam vs. FSol	1.12	0.008027 to 2.232	Yes	*	0.047
FSam vs. FSam+FSol	-0.88	-1.992 to 0.2320	No	ns	0.223
FSam vs. Control	-4.337	-5.449 to -3.225	Yes	***	<.001
FSam vs. FSam	-6.113	-7.225 to -5.001	Yes	***	<.001
FSam vs. FSol	-5.35	-6.462 to -4.238	Yes	***	<.001
FSam vs. FSam+FSol	-8.13	-9.242 to -7.018	Yes	***	<.001
FSol vs. FSam+FSol	-2	-3.112 to -0.8880	Yes	***	<.001
FSol vs. Control	-5.457	-6.569 to -4.345	Yes	***	<.001
FSol vs. FSam	-7.233	-8.345 to -6.121	Yes	***	<.001
FSol vs. FSol	-6.47	-7.582 to -5.358	Yes	***	<.001
FSol vs. FSam+FSol	-9.25	-10.36 to -8.138	Yes	***	<.001
FSam+FSol vs. Control	-3.457	-4.569 to -2.345	Yes	***	<.001
FSam+FSol vs. FSam	-5.233	-6.345 to -4.121	Yes	***	<.001
FSam+FSol vs. FSol	-4.47	-5.582 to -3.358	Yes	***	<.001
FSam+FSol vs. FSam+FSol	-7.25	-8.362 to -6.138	Yes	***	<.001

Control vs. FSam	-1.777	-2.889 to - 0.6647	Yes	***	<.001
Control vs. FSol	-1.013	-2.125 to 0.09864	No	ns	0.1
Control vs. FSam+FSol	-3.793	-4.905 to -2.681	Yes	***	<.001
FSam vs. FSol	0.7633	-0.3486 to 1.875	No	ns	0.395
FSam vs. FSam+FSol	-2.017	-3.129 to - 0.9047	Yes	***	<.001
FSol vs. FSam+FSol	-2.78	-3.892 to -1.668	Yes	***	<.001
40 d					
Control vs. FSam	-2	-3.112 to - 0.8880	Yes	***	<.001
Control vs. FSol	-1.587	-2.699 to - 0.4747	Yes	***	<.001
Control vs. FSam+FSol	-2.963	-4.075 to -1.851	Yes	***	<.001
Control vs. Control	-5.897	-7.009 to -4.785	Yes	***	<.001
Control vs. FSam	-8.747	-9.859 to -7.635	Yes	***	<.001
Control vs. FSol	-7.197	-8.309 to -6.085	Yes	***	<.001
Control vs. FSam+FSol	-9.79	-10.90 to -8.678	Yes	***	<.001
FSam vs. FSol	0.4133	-0.6986 to 1.525	No	ns	0.939
FSam vs. FSam+FSol	-0.9633	-2.075 to 0.1486	No	ns	0.137
FSam vs. Control	-3.897	-5.009 to -2.785	Yes	***	<.001
FSam vs. FSam	-6.747	-7.859 to -5.635	Yes	***	<.001
FSam vs. FSol	-5.197	-6.309 to -4.085	Yes	***	<.001
FSam vs. FSam+FSol	-7.79	-8.902 to -6.678	Yes	***	<.001
FSol vs. FSam+FSol	-1.377	-2.489 to - 0.2647	Yes	**	0.006
FSol vs. Control	-4.31	-5.422 to -3.198	Yes	***	<.001
FSol vs. FSam	-7.16	-8.272 to -6.048	Yes	***	<.001
FSol vs. FSol	-5.61	-6.722 to -4.498	Yes	***	<.001
FSol vs. FSam+FSol	-8.203	-9.315 to -7.091	Yes	***	<.001
FSam+FSol vs. Control	-2.933	-4.045 to -1.821	Yes	***	<.001
FSam+FSol vs. FSam	-5.783	-6.895 to -4.671	Yes	***	<.001
FSam+FSol vs. FSol	-4.233	-5.345 to -3.121	Yes	***	<.001
FSam+FSol vs. FSam+FSol	-6.827	-7.939 to -5.715	Yes	***	<.001
Control vs. FSam	-2.85	-3.962 to -1.738	Yes	***	<.001
Control vs. FSol	-1.3	-2.412 to - 0.1880	Yes	*	0.011
Control vs. FSam+FSol	-3.893	-5.005 to -2.781	Yes	***	<.001
FSam vs. FSol	1.55	0.4380 to 2.662	Yes	**	0.001
FSam vs. FSam+FSol	-1.043	-2.155 to 0.06864	No	ns	0.081
FSol vs. FSam+FSol	-2.593	-3.705 to -1.481	Yes	***	<.001

60 d					
Control vs. FSam	-1.94	-3.052 to -0.8280	Yes	***	<.001
Control vs. FSol	-1.213	-2.325 to -0.1014	Yes	*	0.023
Control vs. FSam+FSol	-2.737	-3.849 to -1.625	Yes	***	<.001
Control vs. Control	-5.11	-6.222 to -3.998	Yes	***	<.001
Control vs. FSam	-7.787	-8.899 to -6.675	Yes	***	<.001
Control vs. FSol	-7.3	-8.412 to -6.188	Yes	***	<.001
Control vs. FSam+FSol	-8.757	-9.869 to -7.645	Yes	***	<.001
FSam vs. FSol	0.7267	-0.3853 to 1.839	No	ns	0.459
FSam vs. FSam+FSol	-0.7967	-1.909 to 0.3153	No	ns	0.34
FSam vs. Control	-3.17	-4.282 to -2.058	Yes	***	<.001
FSam vs. FSam	-5.847	-6.959 to -4.735	Yes	***	<.001
FSam vs. FSol	-5.36	-6.472 to -4.248	Yes	***	<.001
FSam vs. FSam+FSol	-6.817	-7.929 to -5.705	Yes	***	<.001
FSol vs. FSam+FSol	-1.523	-2.635 to -0.4114	Yes	**	0.001
FSol vs. Control	-3.897	-5.009 to -2.785	Yes	***	<.001
FSol vs. FSam	-6.573	-7.685 to -5.461	Yes	***	<.001
FSol vs. FSol	-6.087	-7.199 to -4.975	Yes	***	<.001
FSol vs. FSam+FSol	-7.543	-8.655 to -6.431	Yes	***	<.001
FSam+FSol vs. Control	-2.373	-3.485 to -1.261	Yes	***	<.001
FSam+FSol vs. FSam	-5.05	-6.162 to -3.938	Yes	***	<.001
FSam+FSol vs. FSol	-4.563	-5.675 to -3.451	Yes	***	<.001
FSam+FSol vs. FSam+FSol	-6.02	-7.132 to -4.908	Yes	***	<.001
Control vs. FSam	-2.677	-3.789 to -1.565	Yes	***	<.001
Control vs. FSol	-2.19	-3.302 to -1.078	Yes	***	<.001
Control vs. FSam+FSol	-3.647	-4.759 to -2.535	Yes	***	<.001
FSam vs. FSol	0.4867	-0.6253 to 1.599	No	ns	0.867
FSam vs. FSam+FSol	-0.97	-2.082 to 0.1420	No	ns	0.131
FSol vs. FSam+FSol	-1.457	-2.569 to -0.3447	Yes	**	0.003
